# Increased Ca^2+^ signaling in *NRXN1α*^*+/−*^ neurons derived from ASD induced pluripotent stem cells

**DOI:** 10.1186/s13229-019-0303-3

**Published:** 2019-12-30

**Authors:** Sahar Avazzadeh, Katya McDonagh, Jamie Reilly, Yanqin Wang, Stephanie D. Boomkamp, Veronica McInerney, Janusz Krawczyk, Jacqueline Fitzgerald, Niamh Feerick, Matthew O’Sullivan, Amirhossein Jalali, Eva B. Forman, Sally A. Lynch, Sean Ennis, Nele Cosemans, Hilde Peeters, Peter Dockery, Timothy O’Brien, Leo R. Quinlan, Louise Gallagher, Sanbing Shen

**Affiliations:** 1 Regenerative Medicine Institute, School of Medicine, Biomedical Science Building BMS-1021, National University of Ireland Galway, Dangan, Upper Newcastle, Galway, Ireland; 2 Department of Physiology, College of Life Science, Hebei Normal University, Shijiazhuang, China; 3 HRB Clinical Research Facility, National University of Ireland (NUI), Galway, Ireland; 4 Department of Haematology, Galway University Hospital, Galway, Ireland; 5 School of Medicine, Trinity College Dublin, Dublin, Ireland; 6 School of Medicine, Conway Institute, University College Dublin, Belfield, Dublin 4, Ireland; 7 Children’s University Hospital, Temple Street, Dublin, Ireland; 8 Department of Clinical Genetics, OLCHC, Dublin 12, Ireland; 9 Children’s University Hospital, Temple St, Dublin, Ireland; 10 Academic Center on Rare Diseases, School of Medicine and Medical Science, University College Dublin, Dublin, Ireland; 11 UCD Academic Centre on Rare Diseases, School of Medicine and Medical Science, University College Dublin, Dublin, Ireland; 12 Centre for Human Genetics, University Hospital Leuven, KU Leuven, 3000 Leuven, Belgium; 13 Centre for Microscopy and Imaging, Anatomy, School of Medicine, National University of Ireland (NUI), Galway, Ireland; 14 Physiology and Human Movement Laboratory, CÚRAM SFI Centre for Research in Medical Devices, School of Medicine, National University of Ireland (NUI), Galway, Ireland

**Keywords:** Autism, Calcium signaling, Induced pluripotent stem cells, Neurons, *NRXN1α*, Transcriptome

## Abstract

**Background:**

Autism spectrum disorder (ASD) is a neurodevelopmental disorder with a high co-morbidity of epilepsy and associated with hundreds of rare risk factors. *NRXN1* deletion is among the commonest rare genetic factors shared by ASD, schizophrenia, intellectual disability, epilepsy, and developmental delay. However, how *NRXN1* deletions lead to different clinical symptoms is unknown. Patient-derived cells are essential to investigate the functional consequences of *NRXN1* lesions to human neurons in different diseases.

**Methods:**

Skin biopsies were donated by five healthy donors and three ASD patients carrying *NRXN1α*^*+/−*^ deletions. Seven control and six *NRXN1α*^*+/−*^ iPSC lines were derived and differentiated into day 100 cortical excitatory neurons using dual SMAD inhibition. Calcium (Ca^2+^) imaging was performed using Fluo4-AM, and the properties of Ca^2+^ transients were compared between two groups of neurons. Transcriptome analysis was carried out to undercover molecular pathways associated with *NRXN1α*^*+/−*^ neurons.

**Results:**

*NRXN1α*^*+/−*^ neurons were found to display altered calcium dynamics, with significantly increased frequency, duration, and amplitude of Ca^2+^ transients. Whole genome RNA sequencing also revealed altered ion transport and transporter activity, with upregulated voltage-gated calcium channels as one of the most significant pathways in *NRXN1α*^*+/−*^ neurons identified by STRING and GSEA analyses.

**Conclusions:**

This is the first report to show that human *NRXN1α*^*+/−*^ neurons derived from ASD patients’ iPSCs present novel phenotypes of upregulated VGCCs and increased Ca^2+^ transients, which may facilitate the development of drug screening assays for the treatment of ASD.

## Background

Autism spectrum disorder (ASD) is a chronic neurodevelopmental disorder characterized by repetitive behavior and deficits in social interaction and communication skills. Epilepsy, intellectual disabilities, language delay, anxiety, and hyperactivity are highly comorbid with ASD [1]. An increased ratio of synaptic excitation/inhibition (E/I) affecting neuroplasticity has been proposed as a common pathway for ASD [2]. This has been linked to altered functional and structural connectivity. Additional evidence from post-mortem neuropathology also showed reduced parvalbumin and altered density/abundance of glutamatergic receptors including *GRM5* and *GRIA1* in ASD [3–5]. On the other hand, overproduction of GABAergic neurons with *FOXG1* overexpression and accelerated cell cycle were also reported in induced pluripotent stem cells (iPSCs) of sporadic ASD with macrocephaly [6].

For synaptic excitation, rare mutations in *NRXN*, *NLGN*, and *SHANK* are reported in individuals with ASD and intellectual disability, further supporting the E/I imbalance hypothesis [7]. *NRXN1* and *SHANK2* are in fact the commonest rare genetic factors identified by a meta-analysis of multiple genetic studies [8, 9]. Notably, *NRXN1* deletions are shared by ASD [10–15], schizophrenia [16–20], intellectual disability [21], ADHD [22], and epilepsy [14, 23–26]. Whereas most human deletions involve in 5′ of *NRXN1α*^*+/−*^ with diverse clinical phenotypes, mouse *Nrxn1α*^*−/−*^ mutants display only mild behavioral deficit in nest building but are otherwise viable, fertile, and indistinguishable from wild-type littermates [27]. This suggests that mouse and human may have different sensitivity to *NRXN1α* gene lesions.

Three *NRXN1* family members (*NRXN1-3*) exist in the genome, and *Nrxn1α*^*−/−*^*/Nrxn2α*^*−/−*^*/Nrxn3α*^*−/−*^ triple knockout mice are impaired in Ca^2+^-triggered neurotransmitter release with altered expression of synaptic Ca^2+^ channels and die of lung dysfunction [28]. Ca^2+^ concentration in neurons is tightly controlled by distinct influx/efflux mechanisms. Ca^2+^ influx occurs commonly through voltage-gated calcium channels (VGCCs) on membrane [29, 30], which facilitate a Ca^2+^ rise during neuronal firing. The influx of Ca^2+^ triggers vesicle exocytosis and neurotransmitter release. The long form of Nrnx1α has been shown to couple release-ready vesicles with metabotropic receptors, facilitating Ca^2+^-triggered exocytosis of neurons [31].

In addition to the long NRXN1α isoforms, which interact with post-synaptic Neuroligins and influence both excitation and inhibition through coupling to GABAergic or NMDA/AMPA receptors [31] and VGCCs [32], *NRXN1* also encodes short NRXN1β isoforms by an alternative promoter, which is largely associated with creation of the scaffolding for excitation [33–35]. *NRXN1α* is therefore proposed to influence E/I balance in both directions, whereas *NRXN1β* primarily mediates excitation. Indeed, conditional knockdown of *NRXN1β* severely impaired the neurotransmitter release at excitatory synapses [36]. It is likely that NRXN1*α* deletion may display increased neuronal excitability, as a result of reduced ratio of *NRXN1α* to *NRXN1β* isoforms, and/or a compensatory increase of *NRXN1β* expression if it happens. Pak et al. showed a reduced mEPSC frequency in human ESC-derived neurons after disrupting shared exon 19 or 24 of *NRXN1* gene, which knocked out an entire *NRXN1* allele with all *NRXN1α/β* isoforms [37]. However, this is different from the genetics in the majority of patients who carry heterozygous deletion at 5′ of *NRXN1* gene which affect *NRXN1α* only, and to date, there have been no patient models to investigate the effects of isoform deletion and/or genetic background. Moreover, it has been shown that common pathophysiological social and cognitive deficits in autism can be linked to gain of function of synaptic proteins and ion channels [7]. These include hyperactivity in frontal brain regions, high-frequency oscillation in cortical regions, and the presence of clinically apparent seizures in 30% of autistic individuals [38–42]. In addition, mutation in neuronal adhesion molecule *CNTN5* has also shown hyper-excitability and increased excitation in iPSC-derived neurons of ASD individuals [43]. These studies show the presence of hyper-excitability and hyperactivity in some of the ASD patients.

The iPSC technology now offers significant benefits for disease modeling [44–46], which can be derived from patient somatic tissues. They resemble embryonic stem (ES) cells and can be differentiated into disease cell types, so to provide human models for investigating disease progression and testing therapeutic drugs, in particular for organs such as the brain and heart, which are impossible to culture by conventional methods. We therefore derived iPSCs from controls and ASD patients carrying *NRXN1*α^+/−^ and differentiated them into cortical excitatory neurons, as altered cortical regions, thickness, folding, surface, columnar lamination, and the number excitatory neurons have been reported in ASD [1, 47–51]. We investigated Ca^2+^ signaling and the transcriptome in day 100 neurons and provided novel phenotype with increased Ca^2+^ transients and upregulated VGCCs in ASD *NRXN1α*^+/−^ neurons.

## Methods

### Participants

Ethical approval for the study was obtained from the St. James’s/Tallaght University Hospital and the Galway University Hospital Clinical Research Ethics Committee. Seven control iPSC lines were derived from five healthy donors (Additional file 1: Table S1). The sample 1C was donated by a healthy sibling of patient ND1, the 4C (male), the 2V (female), and the 3V (male) by healthy volunteers. The NCRM1 control line was derived by NIH from a newborn boy.

All patients had confirmed research diagnoses of ASD with the Autism Diagnostic Interview-Revised and the Autism Diagnostic Observational Schedule (Additional file 1: Table S1) [52, 53]. Six *NRXN1α*^*+/−*^ iPSC lines were generated from three ASD patients (Additional file 1: Figure S1A). The ND1 was donated by a non-verbal male with severe intellectual disability, autism, infant seizures, developmental delay, self-injurious and aggressive behavior, and carrying de novo *NRXN1α* ^*+/−*^ deletion on exons 6–15 (chr2:50711687-51044633, Hg19). The ND2 was a male patient carrying *NRXN1α* ^*+/−*^ deletion in exons 1–5 (Chr2:51120335-51360666, Hg19), with autism, language delay, IQ of 78 at age 11, but attended mainstream education. One of ND2’s parents had language delay, and one grandfather and one cousin had ASD. The ND4-1 female was diagnosed with Asperger’s syndrome, social anxiety, psychosis, and mild intellectual disability, with an IQ of 69, a history of seizures, and a paternal *NRXN1α*^*+/−*^ lesion (chr2:50983186-51471321). Her paternal grandmother was institutionalized, and her father and paternal aunt had seizures.

Genomic DNA from parental fibroblasts and iPSC lines was extracted with DNeasy kit (69504, Qiagen). An Illumina 1M SNP array was performed at UCD. All samples passed quality control with call rates > 99%. CNV analysis was carried out using PennCNV. False-positive CNVs were excluded using SNP < 10 or kb < 100. The *NRXN1α* deletions were confirmed (Additional file 1: Figure S1A), and additional putative CNVs detected are listed in Additional file 1: Table S7.

### iPSC derivation

Skin punches were obtained with consent in the Clinical Research Facility. Biopsy was cut, dragged along the rough surface of culture dishes for adherent culture at 37 °C with 5% CO_2_ in high glucose DMEM supplemented with 10% FCS, 1% NEAA, and 1% penicillin/streptomycin. The medium was renewed every 2–3 days. Low passage fibroblasts were reprogrammed to iPSCs (Merck-Millipore, SCR510; Thermo Fisher Scientific, or Epi5™ Episomal iPSC Reprogramming Kit; Invitrogen, A15960) and characterized for expression of alkaline phosphatase, NANOG, OCT4, SOX2, SSEA4, TRA-1-60, TRA-1-81, TUJ1, ASM, and AFP.

### Neuronal differentiation

The iPSCs were seeded at 45,000–50,000 cells/cm^2^, grown to ~ 85% confluency in E8 (Thermo Fisher Scientific, A1517001), and differentiated into neural rosettes for 10–12 days in N2B27 (Thermo Fisher Scientific) with 100 nM LDN193189 (Stem Cell technologies, #72102) and 10 nM SB431542 (Sigma, S4317) [52, 53]. Neural rosettes were passaged, cultured for another 10 days, and then plated onto poly-D-Lysine/laminin-coated 12-well plates, 15-mm coverslips, or ibidi 8-well chambers for terminal differentiation. Cells were maintained in N2B27 (w/o vitamin A) for 6 days and then in N2B27 plus vitamin A until analyses by immunocytochemistry, immunoblotting, calcium imaging, or RNA sequencing, respectively. All phenotypic analyses were performed at day 100 of differentiation according to previous published protocol [53].

### Immunocytochemistry

Cells were fixed in 4% paraformaldehyde, blocked with 0.2% BSA, and incubated with primary antibodies (Additional file 1: Table S8) at 4 °C overnight. They were washed, incubated for 1 h at room temperature with appropriate secondary antibody (Additional file 1: Table S8), and mounted with DAPI. Images were taken under a fluorescence microscope and quantified by ImageJ.

### Calcium imaging

Cultures were washed with artificial cerebrospinal fluid (ACSF), incubated with 2 μM Fluo-4 AM (Thermo Fisher scientific, F14201) in ACSF for 20 min at 37 °C, cultured in normal medium at 37 °C for 20 min, and imaged in warm ACSF in an imaging chamber (Warner Instruments, RC-26GLP) on a Zeiss Axiovert 200 microscope (× 10). Videos were captured with a Hamamatsu ORCA284 at 1 Hz frame rate for 3–5 min and stored as uncompressed image sequences.

Chemicals were added to the ACSF as required, i.e., Na^+^ channel blocker TTX (Alomone Labs T-550), AMPA/Kainate receptor blocker CNQX (Alomone Labs C-140), NMDA receptor blocker DL-AP5 (Alomone Labs D-140), L-type VGCC blocker Nifedipine (Alomone Labs N-120), P/Q-type VGCC blocker agatoxin (Alomone Labs STA-500), glutamate (Sigma, G8415), ionomycin (Sigma I0634), or γ-aminobutyric acid (Sigma A2129). Videos were recorded continuously.

FluoroSNNAP in MATLAB (MathWorks, Inc.) was used to analyze calcium image sequences [52, 53]. Neurons with > 5% fluorescence variations during recording were identified by time-lapse analysis and cell soma defined using batch segmentation. A time-varying fluorescence trace was calculated, transient onset identified, and background noise (ΔF/F < 0.05) determined. The frequency, amplitude, duration, and network synchronicity of spontaneous and evoked calcium transients were analyzed by a coding script in R software.

### Quantitative RT-PCR

RNA was extracted (Qiagen, 74104) and reversely transcribed (Qiagen, 205311). RT-PCR was executed in triplicate with primers listed in Additional file 1: Table S9. The average cycle threshold (Ct) values were calculated in both control and *NRXN1α*^*+/−*^ lines from three technical replicates. All Ct values were normalized to expression of a house-keeping gene (*GAPDH*) as dCt. Relative expression was expressed as 2^–dCt^ over *GAPDH* expression or 2^–ddCt^ over the target gene expression in control fibroblasts for iPSC characterization.

### Transcriptomic analysis

RNASeq was performed by BGI as described previously [54–57] on day 100 cortical neurons from six control iPSC lines of four donors and four *NRXN1α*^*+/−*^ lines of three patients. Transcripts were aligned to GRCH37/hg19, and abundance quantified from the FASTQ in Kallisto (v0.43.1) and presented as transcripts per million (TPM). The two groups were analyzed with false discovery rate (FDR) and adjusted multiple *p* value using the DESeq2 in R. PLS discriminant analysis (PLS-DA) was carried out for supervised clustering, confirming the close clustering among controls and patients. PLS-DA is a supervised method for pattern recognition of unsupervised PCA data and uses the partial least squares (PLS) algorithm to explain and predict the membership of observations to several classes using quantitative or qualitative explanatory variables or parameters [58]. Differentially expressed genes (DEGs) were identified using FDR < 0.05, TPM > 2, > 50% reduction, or > 1.7-fold increase based on TPM ratio and analyzed by STRING and Gene Set Enrichment Analysis (GSEA).

### Statistics

All data were expressed as mean ± SEM. All data were tested for normality using the Shapiro-Wilk normality test. Statistical analysis was performed using the Student *t* test or Mann-Whitney *U* test with a *p* < 0.05.

## Results

### Derivation of iPSCs

**Fig. 1:**
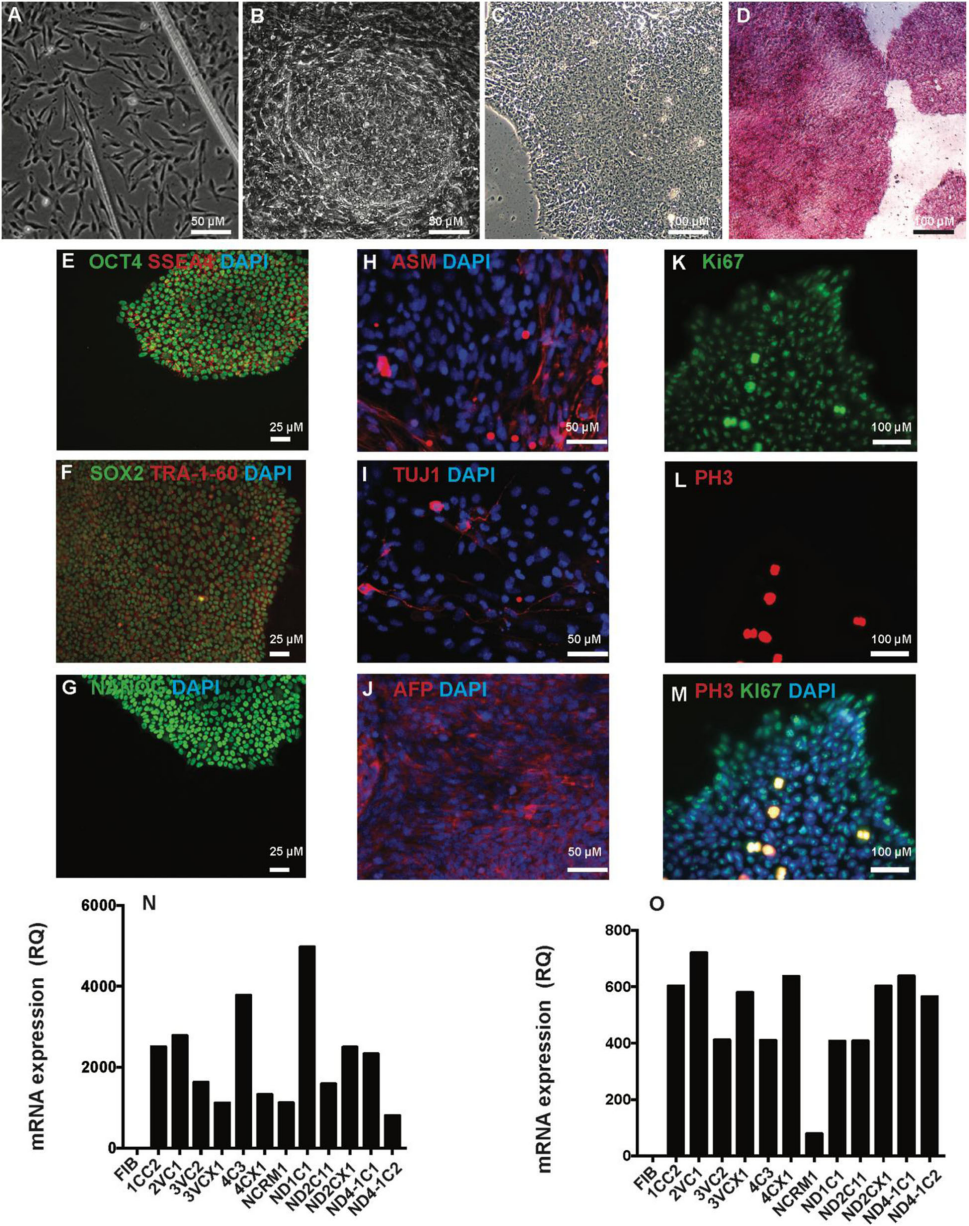
Derivation and validation of iPSCs. **a** Fibroblast outgrowth from the skin biopsy after 12 days of culturing. **b** IPSC colonies were visible and ready for collection after 24 days of reprogramming and became stable after few passaging (**c**). iPSCs were characterized and were stained positive for alkaline phosphatase (**d**) and pluripotent markers OCT4, SOX2, and NANOG and surface markers SSEA4 and TRA-1-60 and TRA-1-81 (**b**–**g**). Spontaneous EB differentiation has shown the expression of markers for mesoderm (ASM, **h**), ectoderm (TUJ1, **i**), and endoderm (AFP, **j**). iPSCs also showed positive expression of proliferating marker Ki67 (**k**) and (**m**) phase marker PH3 (**l**). They were also quantified at mRNA level for the expression of *OCT4* (**n**) and *SOX2* (**o**). All representative images all from control line 4CCX1

In this study, we compared six *NRXN1α*^*+/−*^ iPSC lines from three ASD cases [52, 53] with six iPSC lines from five healthy controls (Additional file 1: Table S1). The mutations were validated by SNP array (Additional file 1: Figure S1A). iPSCs were derived from dermal fibroblasts (Fig. 1a–c) and characterized for pluripotency by expression of alkaline phosphatase (Fig. 1d), NANOG, OCT4, SOX2, SSEA4, and TRA-1-60 (Additional file 1: Figure S2) and tri-germ layer potential by TUJ1, ASM, and AFP (Fig. 1d–j, n, o) and cell cycle markers Ki67 and PH3 (Fig. 1k–m).

### Differentiation of cortical excitatory neurons

We differentiated iPSCs to cortical excitatory neurons using dual SMAD inhibition with LDN193189 and SB431542 [52, 53], and this was accompanied by formation of neural rosettes at 10–12 days, downregulation of *OCT4*, and upregulation of a neural fate marker *PAX6* (Additional file 1: Figure S3A and B). At day 20, 87% of cells were Nestin^+^ and 82% PAX6^+^ (Additional file 1: Figure S3D-F). High levels of expression of *PAX6*, *NES*, *FOXG1*, and *NEUROG2* mRNA demonstrated that they were cortical progenitors (Additional file 1: Figure S3C).

**Fig. 2:**
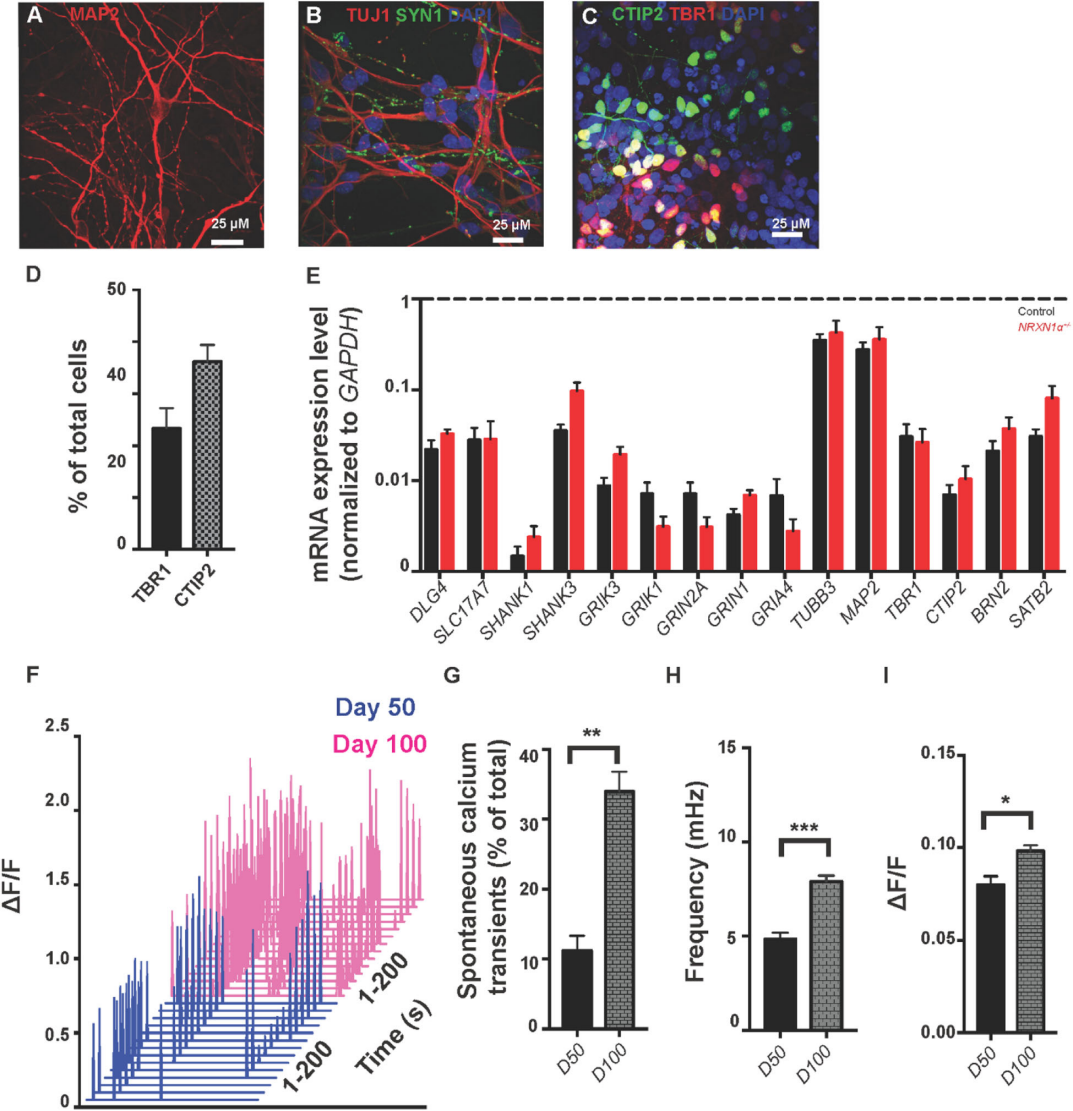
Differentiation and validation of the cortical excitation neurons. **a**–**c** ICC staining of day-100 neurons with MAP2 (**a**), SYN1/TUJ1 (**b**) for neuronal maturity, and with TBR1/CTIP2 for the identity of lower cortical pyramidal neurons (**c**). **d** Proportion of layer VI (TBR1^+^) and layer V/VI (CTIP2^+^) neurons in the cultures. **e** High mRNA expression (normalized to GAPDH [dotted line]) of excitatory post-synaptic markers (*DLG4*, *SHANK1*, *SHANK2*, *SHANK3*, *SLC17A7*, *GRIN1*, *GRIN2A*, *GRIA1*, *GRIA4*, *GRIK1*, *GRIK3*), neuronal markers (*TUBB3*, *MAP2*), upper cortical layers (*BRN2*, *SATB2*), and lower layer markers (*TBR1*, *CTIP2*) in day-100 cultures of control (black) and *NRXN1α*^+/−^ (red) neurons. Results shown from two independent cortical neuronal differentiation (**f**). Representative waterfall traces of spontaneous Ca^2+^ transients in day 50 (blue) and 100 (pink) cultures from 200 s of recording. Neurons exhibited a significant increase in proportion (%) of spontaneous active cells (**g**), the frequency (mHz, **h**), and the amplitude (ΔF/F, **i**) of Ca^2+^ transients from day 50 to day 100. Statistical significance (**p < 0.01, ***p < 0.001, ****p < 0.0001) was evaluated using the Mann-Whitney *U* test). All representative images all from control line 4CCX1

In day 100 cultures of directional differentiation, we compared MAP2^+^ neurons with GFAP^+^ astrocytes and confirmed that the majority of cells were neurons with few astrocytes (Additional file 1: Figure S4B, C), in consistency with the previous publication [53]. There was no significant difference in proportions of MAP2^+^ or GFAP^+^ cells between the controls and *NRXN1α*^*+/−*^ samples, which is different from random differentiation of total *NRXN1* (α and β) knockdown in stem cells (Zeng et al. 2013). The neuronal maturity and synapses were confirmed by positive staining of MAP2/SYN1/TUJ1 (Fig. 2a, b). There was no significant difference in synaptic density and as comparable expression of *MAP2* or *SYN1* mRNA and protein was found in two groups (Additional file 1: Figure S4A, D). In the day 100 culture, 23.4% of cells were layer VI cortical neurons which were positive for TBR1, and 36.2% of cells were layer V-VI neurons expressing CTIP2. RT-PCR showed also equally abundant expression of *BRN2/SATB2* mRNA, the markers for upper-layer neurons. Therefore, the majority of cells were cortical projection neurons (Fig. 2c–e). We examined *NRXN1α* expression by using two pairs of primers derived from exons 9–10 and exons 15–16, respectively, and found 24 or 26% of reduction. Meanwhile, *NRXN1β* expression was increased by 262% as a compensational change (Additional file 1: Figure S7). However, *NRXN2* and *NRXN3* expressions remain unaltered.

High level of postsynaptic excitatory markers (*DLG4*, *SHANK1-3*), vesicular glutamate transporter (*SLC17A7*), inotropic NMDA (*GRIN1*, *GRIN2A*), AMPA (*GRIA1*, *GRIA4*), and Kainate (*GRIK1*, *GRIK3*) receptor mRNA were detected by qRT-PCR (Fig. 2e). RNASeq confirmed extremely low levels of GABAergic (*GABRA1*, *GABRA6*, *GABRD*, *GABRE*, *GABRG3*, *GABRP*, *GABRR1*, and *GABRR2*) gene expression in comparison with excitatory genes (Additional file 1: Figure S5). In addition, GABA (60 μM) elevated Ca^2+^ active cells only by 2.3%, whereas glutamate (60 μM) increased the number of Ca^2+^ active cells by 82.3% (Additional file 1: Figure S6A, B). These data together demonstrated that the majority of day-100 cells in culture were cortical and excitatory neurons.

### Voltage-dependent Ca^2+^ transients

**Fig. 3:**
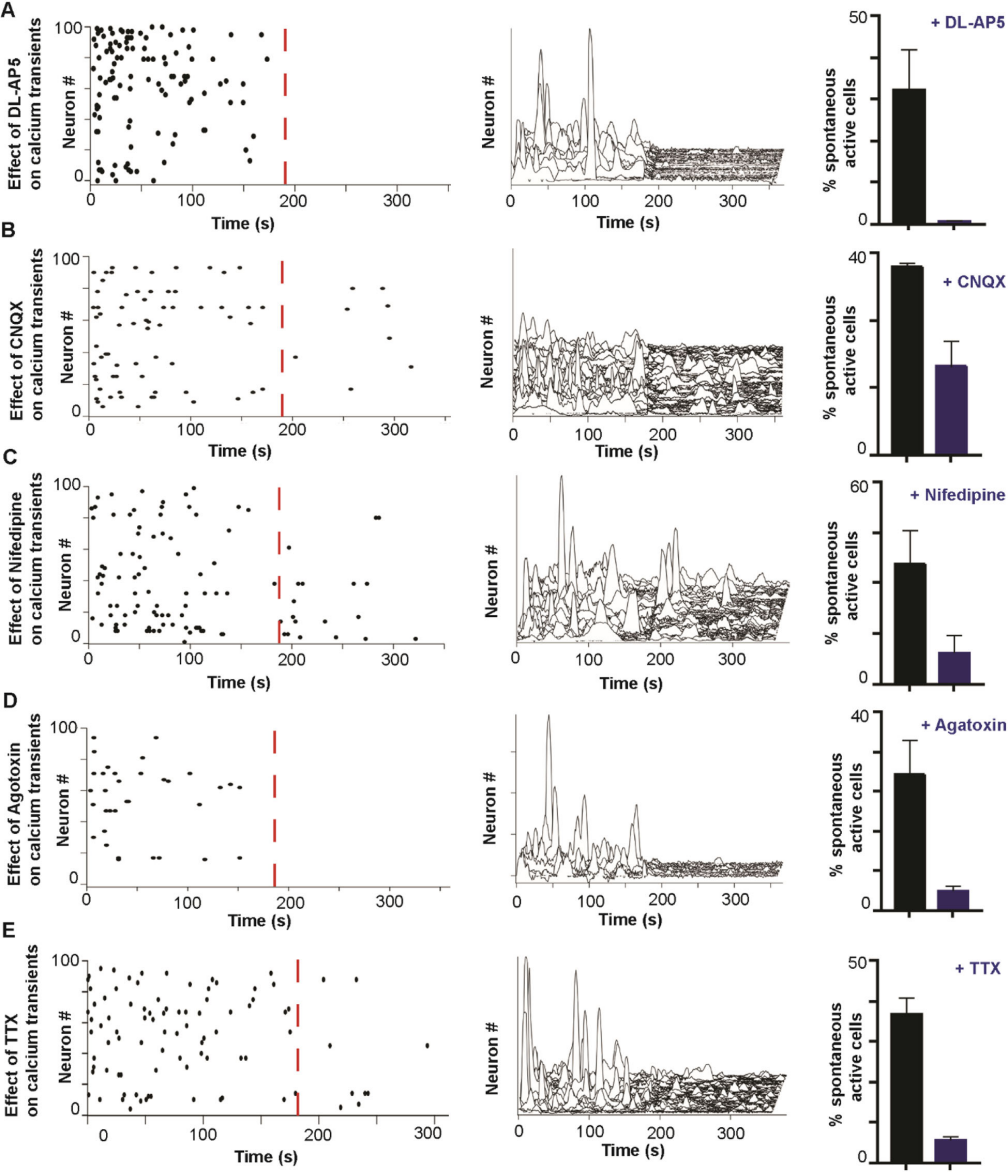
Spontaneous calcium transients are voltage-gated calcium, action potential, and excitatory dependent. **a** Representative raster plots (activity of 100 cells in 300 s of recording, each dot represents Ca^2+^ transient activity, Line 4CX1), waterfall traces (3D representative of calcium transients in 300 s over defined threshold level, Line 4CX1), and their significant change showing the spontaneous Ca^2+^ transient activity of the cells and their response after the application of DL-AP5 (50 μM, **a**) and CNQX (50 μM, **b**), Nifedipine (50 μM, **c**), agatoxin (500 nM, **d**), and TTX (1 μM, **e**) (*n* = 2–3, 1CC1, 3VCX1, 4CX1). The red dotted line shows the point of drug application. All data summary are mean ± SEM

We next validated neuronal functionality by Ca^2+^ imaging (Fig. 2f). The proportion, frequency, and amplitude of spontaneous Ca^2+^ transients were significantly increased from day 50 to 100 (Fig. 2g–i), suggesting that the culture system supported continuous maturation. As the dynamics of calcium signaling can modulate E/I balance through gene regulation and action potential-dependent neurotransmitter release, we analyzed spontaneous Ca^2+^ transient properties in day 100 neurons. The Ca^2+^ transients were shown to be voltage-gated and Na^+^ channel-dependent, as TTX (1 μM) abolished 88% of Ca^2+^ transients (Fig. 3e). DL-AP5 and CNQX reduced Ca^2+^ transients by 98.3% and 61.2%, respectively, suggesting most cells expressed NMDA/AMPA/Kainate receptors (Fig. 3a, b). Nifedipine and agatoxin decreased the number of active cells by 67.3% or 84.0%, suggesting both L- and P/Q-types of VGCCs co-existed in the cultures (Fig. 3c, d). Nifedipine, agatoxin, CNQX, and glutamate showed similar effects on spontaneous calcium transients in *NRXN1α*^*+/−*^ neurons (Additional file 1: Figure S6C). Therefore, spontaneous Ca^2+^ transients in day-100 excitatory neurons are associated with membrane depolarizations (inhibited by TTX) and are facilitated by VGCC (inhibited by nifedipine and agatoxin).

### *NRXN1α*^*+/−*^ deletion altered the kinetics of spontaneous Ca^2+^ transients

**Fig. 4:**
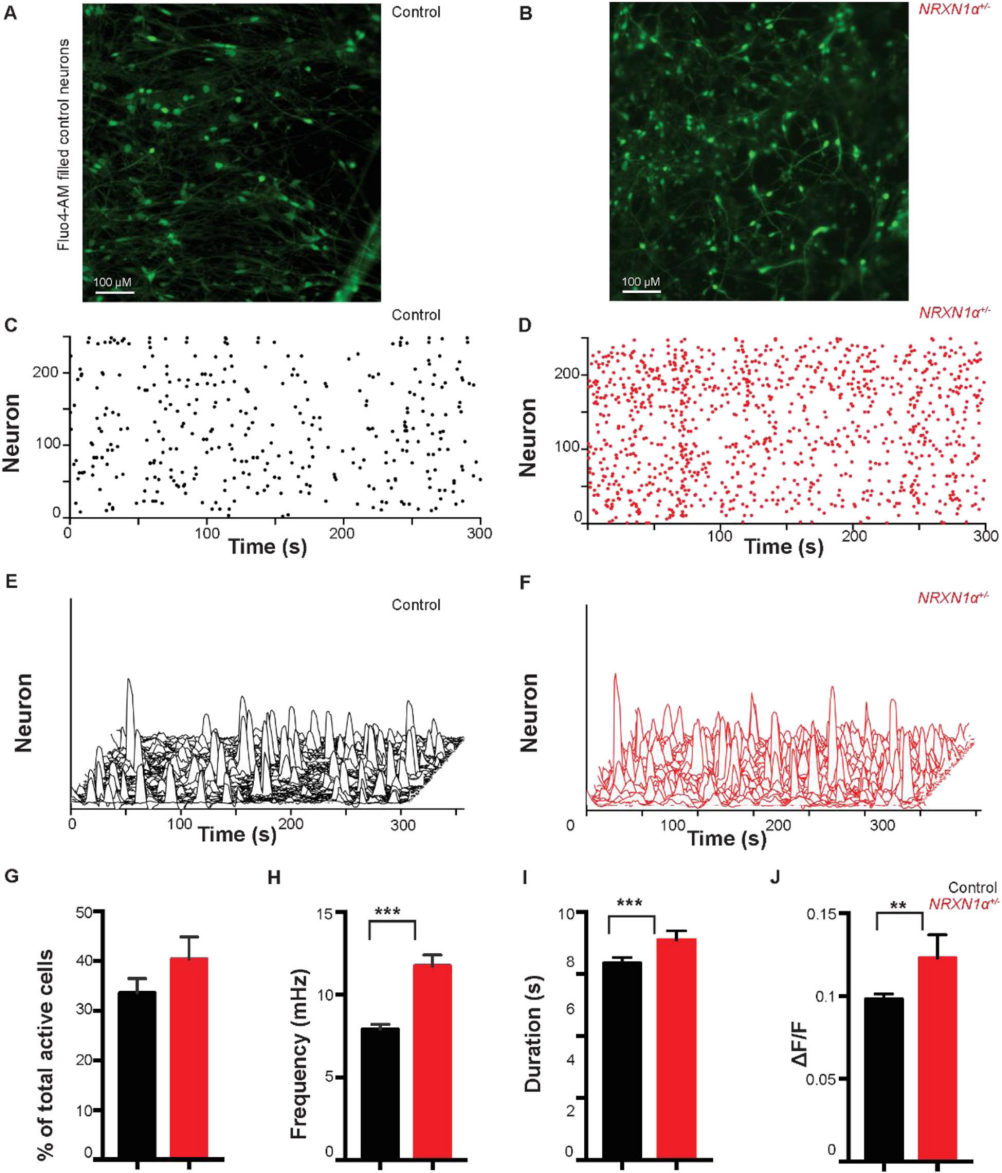
Spontaneous Ca^2+^ transient properties were significantly altered in the day 100 *NRXN1α* ^+/−^ neurons. **a**, **b** The representative images of control (**a**) and patient (**b**) cells loaded with Fluo-4 AM, displaying dense network of neurons in day 100 neuronal cultures. **c**, **d** Representative raster plots showed the spontaneous Ca^2+^ transient activity of the cells from control (**c**) and patient (**d**) cultures. **e**, **f** Representative waterfall traces of spontaneous Ca^2+^ transients in 250 cells over 300 s of recording. **g** The percentage of spontaneous Ca^2+^ transients remained unchanged between the two groups. **h** The frequency of spontaneous Ca^2+^ transients was significantly increased in the *NRXN1α*^*+/−*^ deletion patient cells. **i** The duration of calcium transients was significantly longer in the *NRXN1α*^*+/−*^ deletion patient cells. **j** The amplitude of spontaneous Ca^2+^ transient was significantly increased in the *NRXN1α*^*+/*^ deletion patient cells. Control *n* = 74 recordings/26 coverslips from 6 control iPSC lines (patient *n* = 47 recordings/21 coverslips) from 6 *NRXN1α* ^+/−^ iPSC lines (Additional file 1: Table S10). All data summary are mean ± SEM. Statistical significance (**p < 0.01, ***p < 0.001, ****p < 0.0001) was evaluated using the Mann-Whitney *U* test. Bar = 100 μm in **a**, **b**. Representative images are from control line 4CCX1 and patient ND1C1

We subsequently compared Ca^2+^ transient characteristics in 34,746 control neurons and 19,261 *NRXN1α*^*+/−*^ neurons (Fig. 4a–f) (Additional file1: Figure S8). The regions of interest (neurons) were quantified by batch segmentation within FluoroSNNAP. The proportion of spontaneous active cells was not significantly different between the two groups (control 33.95 ± 2.9%, patient 34.9 ± 2.8%, *p* = 0.92; Fig. 4g). However, the frequency of Ca^2+^ transients was increased by 30.8% in *NRXN1α*^*+/−*^ neurons (11.72 ± 0.7 mHz, *p* < 0.0001) compared to controls (7.91 ± 0.3 mHz, Fig. 4c–f, h). Additionally, the duration of the Ca^2+^ transients was significantly prolonged in *NRXN1α*^*+/−*^ neurons (7.10 ± 0.3 s, *p* = 0.038) versus controls (6.30 ± 0.2 s, Fig. 4i). The amplitude of Ca^2+^ transients was also significantly elevated from controls (0.098 ± 0.003 ΔF/F, Fig. 4j) to *NRXN1α*^*+/−*^ neurons (0.123 ± 0.014 ΔF/F, *p* = 0.008). Therefore, *NRXN1α* deletion significantly altered Ca^2+^ transient characteristics in iPSC-derived cortical excitatory neurons. In addition, all probands were separately investigated against any available family control (Additional file 1: Figure S9) or all controls (Additional file 1: Figure S10). The effect of history of seizures in two probands (ND1, ND4) was also measured in comparison with ND2 (Additional file 1: Figure S11). ASD patient samples showed consistent changes in the frequency of Ca^2+^ transients (Additional file 1: Figure S10, S11).

To address potential variability, we applied separate transformations to the parameters of “amplitude,” “duration,” and “frequency” of Ca^2+^ transients, as our data were non-parametric. We next carried out multivariant analysis of variance (MANOVA) between *NRXN1α*^*+/−*^ and control groups and validated a significant difference between the two groups (Additional file 1: Table S2A). Subsequently, we used two-way nested ANOVA to test each variable on the transformed data, which demonstrated significant differences for “amplitude,” “duration,” or “frequency” between the two groups (Additional file 1: Table S2B).

### VGCC pathway was disrupted in *NRXN1α*^+/−^ neurons

**Fig. 5:**
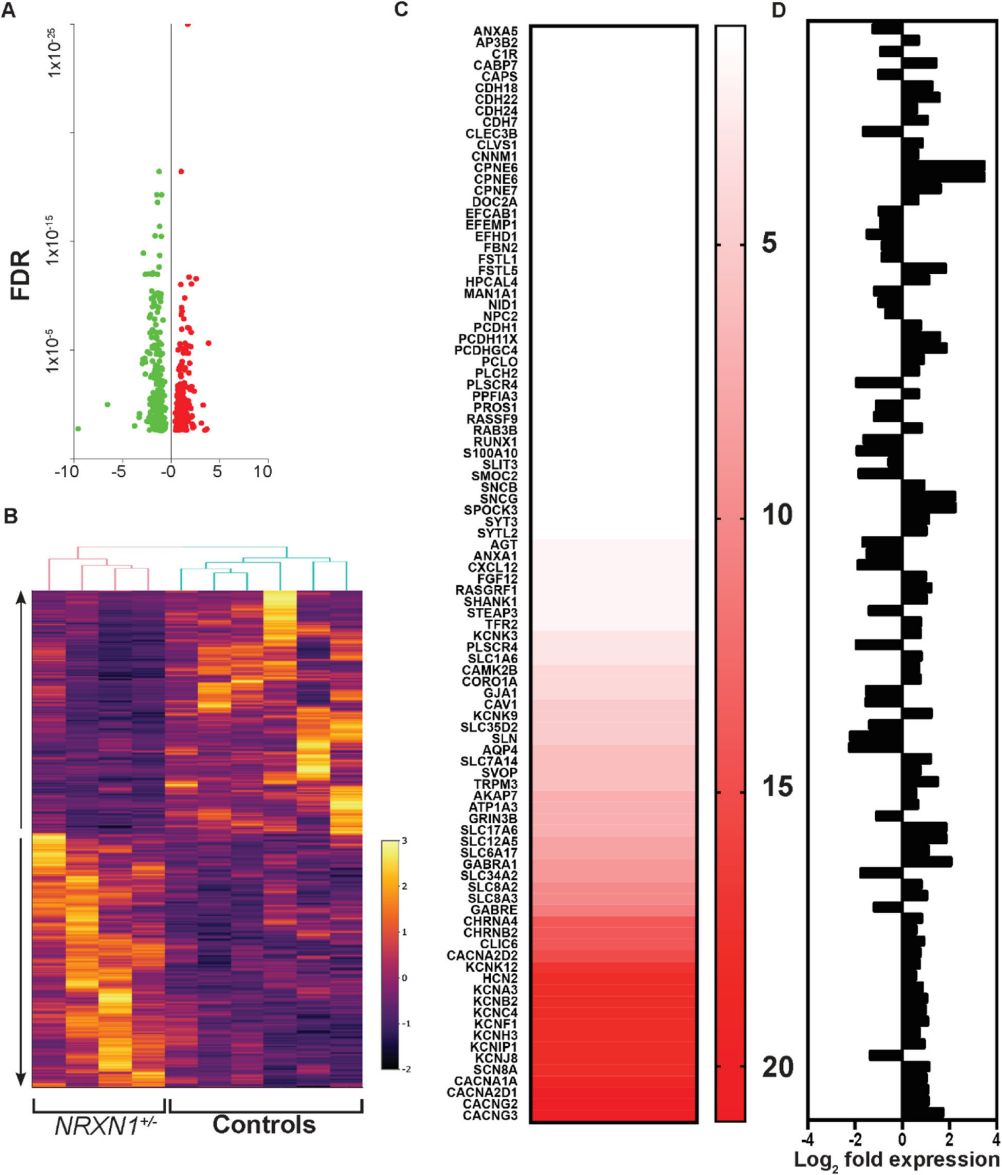
The pathways of calcium and ion transport activity were significantly enriched in *NRXN1α*^***+/−***^ iPSC-derived neurons. **a** Volcano plot of FDR on the *y*-axis and Log_2_ fold changes on the *x*-axis of DEGs in *NRXN1α*^+/−^ neurons. Among the 530 DEGs, 254 were downregulated (in green) and 276 upregulated (in red) with FDR < 0.05. **b** Bivariate clustering of samples (columns) of the 530 DEGs. The color ranges from purple (low expression) to yellow (high expression) based on TPM values (http://rpubs.com/saharava/BivariateClustering). The arrows represent the up/downregulated genes in *NRXN1α*^+/−^ neurons. **c** The heatmap represented all genes which were linked to the 21 pathways. The color showed the most overlapped genes in dark red to non-overlapped genes in white among the pathways. **d** Upregulated (right) and downregulated (left) genes were shown with Log_2_ fold expression. The VGCCs of *CACNA1A*, *CACNA2D1*, and *CACNG2/3* were shared by most pathways

To explore the molecular pathways associated with *NRXN1α*^*+/−*^ ASD neurons, we performed whole-genome RNASeq in day-100 neurons from six controls and four *NRXN1α*^*+/−*^ lines. A total of 27,163 transcripts were quantitatively sequenced and 530 differentially expressed genes (DEGs) identified, with 254 downregulated and 276 upregulated genes (Fig. 5a, b, Additional file 1 Table S3). PLS discriminant analysis (PLS-DA) was carried out for supervised clustering, confirming the close clustering among controls and patients (Additional file 1: Figure S12A). In addition, the expression of DEGs among control and patient iPSC-derived neurons is shown to be consistent (Additional file 1: Figure S12B).

**Fig. 6:**
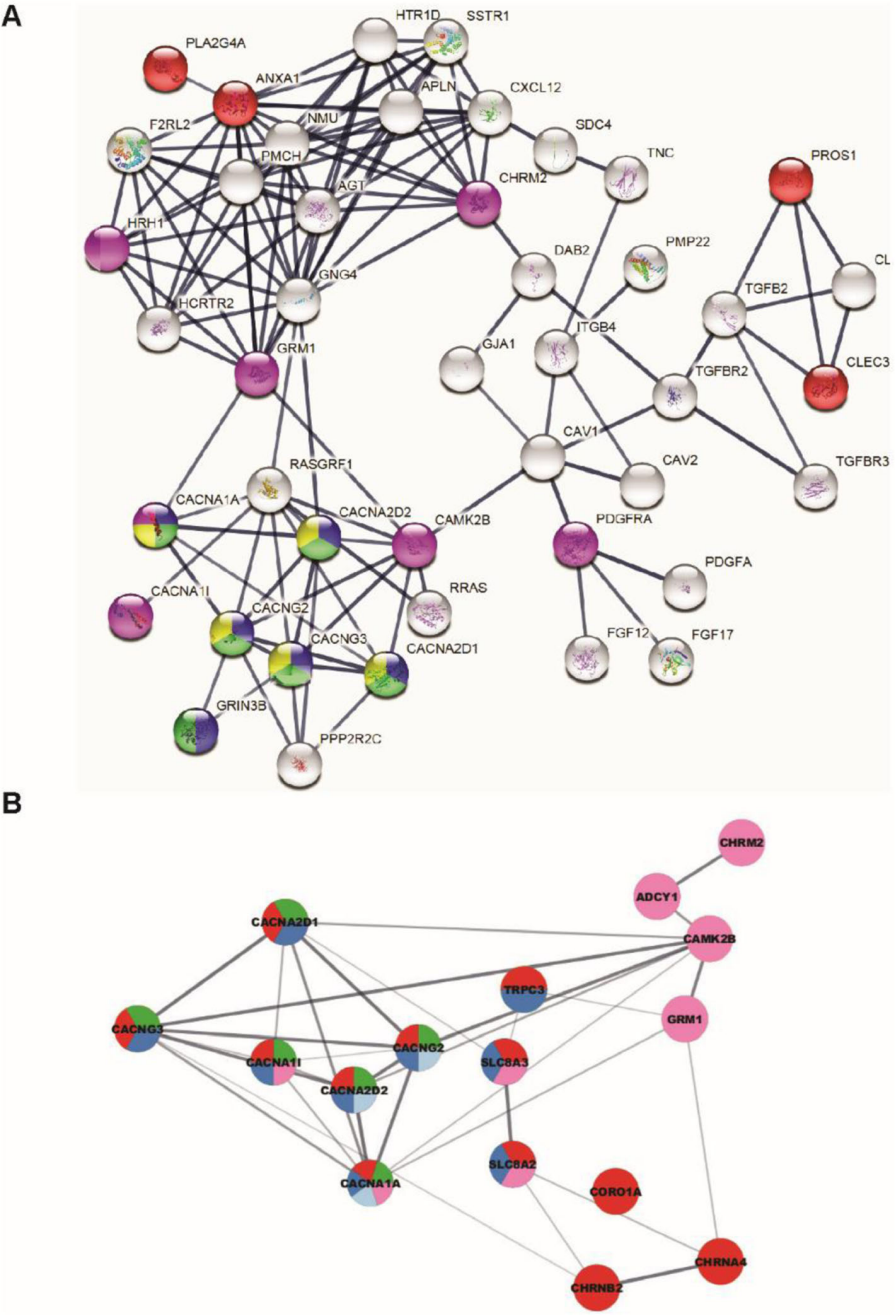
Strong association of calcium channel activity in *NRXN1α*^***+/−***^ iPSC-derived neurons. **a** String KEGG analyses showing a significant network of VGCC association. Proteins in red are associated with calcium ion binding, in blue nodes with calcium ion transmembrane transporter activity, in green node with calcium channel activity, in yellow with voltage-gated calcium channel activity, and in purple with calcium signaling pathway. **b** STRING analyses of upregulated (not downregulated) genes in the *NRXN1α*^*+/−*^ neurons showed association with “calcium ion transport” (red, 12 genes, FDR + 3.20E−03), “calcium ion transmembrane transport” (blue, 9 genes, FDR = 2.06E−02), “voltage-gated calcium channel activity” (green, 6 genes, FDR5.50E−04), calcium signaling pathway (pink, 8 genes, FDR = 4.89E−02), and “presynaptic depolarization and calcium channel opening” (light blue, 3 genes, FDR = 2.37E−02)

STRING and GSEA analyses revealed impairments in calcium binding (GO.0005509, FDR = 7.30E−06), ion transport (GO.0006816, FDR = 7.78E−03), transporter activity (GO.0015085, FDR = 4.92E−02), and voltage-gated channel complexes (GO.0005891, FDR = 2.65E−02) in *NRXN1α*^*+/−*^ neurons (Additional file 1: Table S4). Four VGCC genes, *CACNA1A* (encoding P/Q-type), *CACNA2D1* (encoding L-type), *CACNG2*, and *CACNG3* (encoding auxiliary subunits), were among the most enriched targets in functional pathways (Fig. 5c, Fig. 6a) and were upregulated by 2.02, 1.90, 2.13, and 3.29-fold (Fig. 5d), respectively. Among the 530 targets, *CACNA1A* was identified as the most overlapped gene among the top 20 pathways (Additional file 1: Figure S13). Subsequent STRING analyses of downregulated or upregulated DEGs, respectively, demonstrated exclusive association of calcium signaling pathways with the upregulated (not downregulated) DEGs (Fig. 6b, Additional file 1: Table S5). Taken together, both functional and transcriptome analyses suggest an increase in calcium signaling pathways as a major phenotype in the *NRXN1α*^+/−^ ASD neurons.

## Discussion

*NRXN1*^*+/−*^ deletions are the most frequent single-gene disruptions associated with ASD [10, 12, 14, 15, 59, 60], schizophrenia [16–20], intellectual disability [21], ADHD [22], and epilepsy [14, 23–26]. Little is known about the consequences of *NRXN1*^+/−^ lesions in patients’ neurons or why the same heterozygous *NRXN1*^+/−^ deletions lead to diverse clinical phenotypes. We are the first to report derivation of human iPSCs from ASD patients carrying *NRXN1α*^*+/−*^. The cortical excitatory neurons from *NRXN1α*^*+/−*^ iPSCs displayed a novel phenotype of increased frequency, duration, and amplitude of Ca^2+^ transients. This is supported by transcriptome analyses, which revealed an upregulation of VGCCs (*CACNA1A*, *CACNA2D1*, *CACNG2*, and *CACNG3*) and Ca^2+^ pathways in *NRXN1α*^*+/−*^ neurons.

Typically in neurons, calcium influx is facilitated by the opening of the α1 subunit in the tetrameric VGCCs in response to membrane depolarizations. The α1 subunit is encoded by *CACNA1A*, *CACNA1B*, *CACNA1C*, *CACNA1D*, *CACNA1E*, and *CACNA1S* genes. Consistent with ASD *NRXN1α*^*+/−*^ phenotype, gain-of-function of VGCCs are implicated in neurodevelopmental disorders (Additional file 1: Table S6). For example, Cav1.2 G406R (*CACNA1C*) causes Timothy syndrome with ASD by delayed inactivation and prolonged opening [61, 62]. Knock-in of the G406R to mice results in autistic phenotype [61, 62]. Exome sequencing has identified various *CACNA1D* mutations (encoding Cav1.3) in ASD [63–66], epilepsy [67], and developmental delay [67]. A *CACNA1D* paralog, *CACNA1F* (Cav1.4), also is linked to New Zealand autistic males with excessive Ca^2+^ influx [61, 62].

We have identified *CACNA1A* encoding P/Q-type and *CACNA2D1* encoding L-type VGCC as the most interactive *NRXN1α*^+/−^ targets. *CACNA1A* is predominantly expressed in neurons and involved in NRXN1α signaling which triggers the release of fusion-ready vesicles [68]. *CACNA1A* polymorphisms are associated with Chinese ASD [68], and *CACNA1A* mutations with epileptic encephalopathy [68]. Additionally, mutations in other VGCCs are also identified as a major pathway in schizophrenia [68, 69], the common risks across seven brain diseases [70, 71], and in ASD (Additional file 1: Table S6) [72, 73]. In addition, loss-of-function mutations in some VGCCs are also reported, i.e., *CACNA1H* R212C, R902W, W962C, and A1874V reduce their activity in ASD [74]; *CACNA2D1* is deleted in epilepsy and intellectual disability [74]; *CACNG2* V143L decreases its binding to GLUR1 or GLUR2 [75]; and *Cacng2* hypomorph results in epileptic phenotype [74]. This evidence supports altered VGCCs as a mechanism in ASD *NRXN1α*^*+/−*^ neurons.

The human *NRXN1α*^*+/−*^ phenotype reported here differs from some of the data reported previously. Pak et al. created a mutant human H1 ES cell line with disruption of exon 19 or 24, which are shared by all *NRXN1* isoforms (Additional file 1: Figure S1A), and showed reduced frequency of mEPSCs [37]. *NRXN1* consists of 2 promoters and 11 differentially spliced exons which may result in 2048 *NRXN1α* and 4 *NRXN1β* isoforms. The human H1 ES cells (*NRXN1*^+/−^) from Pak et al. are genetically different from the ASD patients here, who carry 1 copy of *NRXN1α*^+/−^. However, qRT-PCR using primer pairs from exons 9–10 or 15–16 demonstrate 24 or 26% (not 50%) reduction. This is likely due to the complex exon usage of differential NRXN1 splicing. Meanwhile, we observe 262% compensational increase in *NRXN1β* expression; therefore, the phenotype in this study is likely to result from combinational effects of reduced *NRXN1α* and overexpression of *NRXN1β*. This may also re-enforce the concept that *NRXN1α* and *NRXN1β* isoforms play differential roles in neuronal E/I.

Sudhoff et al. propose that Neurexin variants from alternative splicing may perform the same canonical functions but may have different patterns of redundancy [76–78]. *Nrxn1α* homozygous knockout presented no apparent phenotype, and Pak et al. also showed that mouse *Nrxn1* knockout cells differed from H1 ES cells and displayed no phenotype [79]. Mice with triple knockout of *Nrxn1α*, *Nrxn2α* and *Nrxn3α* genes were shown to produce different phenotypes in different neurons or synapses [76, 77]. In hippocampal presynaptic cells, the Ca^2+^ influx was reduced in conjunction with lower Cav2.1-mediated transients and elevated axonal mobility of α2δ1 [80]. Although overexpression of *Nrxn1α* and α2δ1 is shown to rescue Ca^2+^ currents in *Nrxn1α*^*−/−*^*Nrxn2α*^*−/−*^*Nrxn3α*^*−/−*^ triple knockout mouse neurons, this is yet to be investigated in human cells [80]. In addition, species differences also exist: i.e., Nrxn1 at *Caenorhabditis elegans* acetylcholine neuromuscular synapse is located postsynaptically, not presynaptically [32], and approximately > 20% of human essential genes are nonessential in mice [37].

The penetrance of human *NRXN1a*^*+/−*^ is not 100%, and clinical conditions of *NRXN1a*^*+/−*^ are diverse. Therefore, co-factors in the genetic background may play a part in clinical phenotype. Investigations of patient-derived samples are essential for understanding roles of *NRXN1a*^*+/−*^ in different human conditions. The ASD *NRXN1a*^*+/−*^ phenotype here is consistent with the proposal that NRXN1β triggers excitation, and NRXN1α regulates both excitation and inhibition [33–35]. *NRXN1α* deletions are therefore anticipated to weaken neuronal inhibition and increase excitation. A recent publication has shown that ASD neurons derived from autism CNTN5^+/−^ or EHMT2^+/−^ human iPSCs develop hyperactive neuronal networks [43]. This suggests indirect effects of NRXN1α on Ca^2+^ transients. The upregulated CACNA1A, CACNA2D2, and CACNG2 are linked to “the presynaptic depolarization and calcium channel opening” by STRING (Additional file 1: Table S5). Direct interactions of NRXNs with VGCCs are reported but limited. Mouse Nrxn1α is shown to positively modulate Ca^2+^ influx through Cav2.1-α2δ1 interaction [80]. On the other hand, human NRXN1α may also form NRXN1α-Cav2.2-αδ3 complex and negatively regulate Cav2.2 currents in transfected cells [32]. Furthermore, Neuroligins contain Ca^2+^-binding EF-hand domains, and Neuroligin-NRXN1β interaction is dependent on Ca^2+^ [81]. Elevated Ca^2+^ transients in human *NRXN1α*^*+/−*^ neurons may therefore also enhance excitation through increased Neuroligin-NRXN1β interactions. Furthermore, we have observed an increase in the expression of few members of SNARE complexes, i.e., synaptotagmins, suggesting an interaction of the cytoplasmic membrane of neurexins with synaptotagmins [82]. It seems likely that NRXN1α may regulate the level of synaptotagmins or other members of SNARE proteins, which might be critical for neurotransmitter and vesicle release [83]. Interestingly, two of our ASD patients had a history of seizures. While the patient numbers were small, it appeared that the increase of the frequency was more prominent in two ASD probands with seizure (ND1, ND4) than the ASD without seizure (ND2, Additional file 1: Figure S11). This concurs with disrupted Ca^2+^ signaling implicated in a range of neurodevelopmental disorders including ASD and epilepsy [79, 84–88].

The DEGs in *NRXN1α*^*+/−*^ neurons may arise from Ca^2+^ influx and voltage-dependent conformational changes of VGCCs. For example, Cav1.2 may interact with αCaMKII, and βCaMKII is then recruited by Ca^2+^ mobilization. Voltage-dependent conformational changes can lead to α/βCaMKII activation, CREB phosphorylation and nuclear accumulation [89], and activation of transcription factors NFAT and MEF2 [90–94]. Therefore, the transcriptomic changes may reflect both the activity-driven alterations and functional features of ASD *NRXN1α*^+/−^ neurons.

## Limitations

There are several limitations which may be addressed in the follow-up studies. (1) While we provide strong evidence for the role of VGCCs as a contributor to alterations in NRXN1α^+/−^ neurons, in this study, we employed the non-ratiometric calcium reporter Fluo-4 AM to represent intracellular calcium dynamics in the absence of ground-truth electrophysiological recordings and direct measurements of VGCCs. Future studies will be required to directly measure the channel activation and kinetics in NRXN1α^+/−^ neurons. (2) The NRXN1 deletions are associated with different clinical symptoms; therefore, NRXN1 deletion iPSCs from different neurodevelopmental/neuropsychiatric diseases may be investigated through collaborative research (3). The heterogeneity of iPSCs is common. Although the current data are conducted with statistically viable numbers and vigorously justified with different statistical methods, experiments with a larger cohort of iPSC lines will be desirable to confirm the commonality of the phenotype. (4) Genetic rescue will be important to validate genotype-phenotype correlation, but this is technically challenging, given that the *NRXN1* deletion sizes of chromosomal regions are beyond the limit of conventional rescue constructs. In addition, the NRXN1 non-coding sequences are evolutionally conserved, and NRXN1 gene expression is highly regulated; therefore, no single cDNA-based construct may be able to rescue the phenotype with the right dose, isoform, and/or developmental regulation of the NRXN1 expression. (5) As the clinical penetrance of NRXN1 deletion is incomplete, a second hit may be required for different clinical phenotypes. Creation of isogenic lines with large chromosomal deletions is under the way, albeit technically challenging. It remains to see if the isogenic lines on healthy genetic background will have the same cellular phenotype as from the ASD individuals.

## Conclusions

*NRXN1α*^*+/−*^ neurons derived from ASD patients’ iPSCs revealed alteration in calcium transients’ properties, leading to increased calcium activity. These findings may suggest an alteration in neurotransmitter release and a possible higher excitability in neurons. The *NRXN1α*^+/−^ iPSCs may be offered as a human model with translatable phenotype for drug screening and testing of ASD.

## Supplementary information

**Additional file 1.** Supplementary figures and tables.

## Data Availability

Data are available on request from the corresponding author.

## Abbreviations

ASD: Autism spectrum disorder
E/I: Excitation/inhibition
iPSC: Induced pluripotent stem cell
NRXN: Neurexin
VGCC: Voltage-gated calcium channel
