# Supplementary material for: Increased Ca2+ signaling in NRXN1α+/− neurons derived from ASD induced pluripotent stem cells
Source: Mol Autism. 2019 Dec 30;10:52. doi: 10.1186/s13229-019-0303-3 (PMC6937972; doi:10.1186/s13229-019-0303-3)
Supplement: Supplementary file 1 — Additional file 1. Supplementary figures and tables. [file 13229_2019_303_MOESM1_ESM.docx]

**Additional file 1: supplementary tables + figures**

Table 1. Source information for iPSC lines.

| **iPSC line** | **Reprog.**  **method** | **Control/**  **patient** | **Sex** | **Biopsy**  **Age** | **Inheritance** | **Deletion region (Hg19)** |
| --- | --- | --- | --- | --- | --- | --- |
| 1CC2 | Lentivirus | Control | M | 4 | N/A | N/A |
| 3VCX1, 3VC2 | Lentivirus | Control | M | 21 | N/A | N/A |
| 4CC3, 4CCX1 | Lentivirus | Control | M | 19 | N/A | N/A |
| 2VC1 | Lentivirus | Control | F | 20 | N/A | N/A |
| NCRM-1 (NIH) | Episomal | Control | M | <1 | N/A | N/A |
| ND1C1, ND1C4 | Lentivirus | ASD Patient | M | 8 | De novo | chr2:50711687-51044633 |
| ND2C11, ND2CX1 | Lentivirus | ASD Patient | M | 20 | De novo | chr2:51120335-51360666 |
| ND4-1C1, ND4-1C2 | Episomal | ASD Patient | F | 18 | Paternal | chr2:50983186-51471321 |

**Table 2A**: The summary of the MANOVA test on control and NRXN1α+/- groups on calcium signaling

|  | **Df** | **F statistics** | **P-value** |
| --- | --- | --- | --- |
| NRXN1α^+/-^ vs Control | 1 | 63.828 | < 0.001 |
| Cohort* | 8 | 4.156 | < 0.001 |
| Residuals | 98 |  |  |

**Table 2B**. The summary of Two-way ANOVA test on Amplitude, Duration and Frequency of calcium transients.

| **NRXN1α^+/-^ vs Control** | **F statistics** | **P-value** |
| --- | --- | --- |
| Amplitude | 6.10 | 0.015 |
| Duration | 14.18 | <0.001 |
| Frequency | 156.68 | <0.001 |

**Table 3. Differentially expressed genes (DEGs) between the control (black) and NRXN1α^+/-^ lines (red). (in Excel file)** The transcripts in six control lines (NCRM1, 2VC1, 3VC1, 3VCX1 4CC3, 4CCX1 in black) and four NRXN1α^+/-^ (ND1C1, ND2C11, ND2CX1, ND4-1C1 in red) lines were quantified by RNASeq. Gene expression are presented in Transcripts Per Million (TPM). The significance was indicated by FDR (<0.05) from R program. The Mean in Control and in NRXN1α^+/-^ lines, the fold of changes (NRXN1α^+/-^ /Control) were calculated. The DEGs are arranged in alphabetic order for down-regulated genes and then up-regulated genes.

| NCRM1 | 2vc1 | 3vc2 | 3vcx1 | 4c3 | 4cx1 | ND2_11 | ND2_X1 | ND4_2 | ND1_c1 | **ConTPM** | **ND TPM** | ratio | (FDR) P adj | JR symbol |
| --- | --- | --- | --- | --- | --- | --- | --- | --- | --- | --- | --- | --- | --- | --- |
| **17.04** | **21.48** | **16.80** | **10.73** | **26.32** | **19.51** | **52.11** | **26.89** | **52.50** | **48.09** | **18.65** | **44.90** | 2.41 | **1.735E-03** | **ABLIM3** |
| **3.06** | **3.64** | **1.16** | **1.31** | **0.98** | **2.44** | **3.09** | **4.11** | **8.68** | **5.49** | **2.10** | **5.34** | 2.54 | **6.713E-05** | **ACTG1P4** |
| **33.90** | **37.64** | **47.98** | **66.19** | **88.59** | **25.22** | **101.42** | **105.83** | **99.15** | **67.79** | **49.92** | **93.55** | 1.87 | **1.676E-02** | **ACTL6B** |
| **4.04** | **3.37** | **2.00** | **3.47** | **3.59** | **1.78** | **5.18** | **5.90** | **6.72** | **5.05** | **3.04** | **5.71** | 1.88 | **4.276E-02** | **ADAM1A** |
| **10.68** | **16.15** | **11.49** | **12.34** | **21.90** | **13.08** | **41.74** | **25.23** | **36.21** | **26.24** | **14.27** | **32.36** | 2.27 | **4.286E-03** | **ADAP1** |
| **2.59** | **3.07** | **1.16** | **0.91** | **0.47** | **1.42** | **3.75** | **1.78** | **8.49** | **1.91** | **1.60** | **3.98** | 2.48 | **2.643E-02** | **ADARB2** |
| **28.55** | **43.65** | **41.28** | **34.74** | **44.30** | **54.40** | **94.72** | **57.44** | **93.63** | **58.99** | **41.15** | **76.20** | 1.85 | **2.295E-02** | **ADCY1** |
| **16.87** | **23.01** | **15.92** | **15.86** | **22.41** | **21.97** | **48.10** | **38.29** | **36.10** | **24.36** | **19.34** | **36.71** | 1.90 | **1.655E-02** | **ADGRB1** |
| **7.96** | **10.40** | **5.61** | **10.32** | **10.30** | **6.15** | **43.46** | **23.01** | **24.58** | **16.82** | **8.46** | **26.97** | 3.19 | **3.667E-07** | **AFDN-AS1** |
| **0.00** | **0.10** | **1.10** | **0.29** | **0.76** | **1.07** | **5.31** | **1.59** | **3.84** | **0.99** | **0.55** | **2.93** | 5.30 | **3.343E-03** | **AKAIN1** |
| **11.34** | **16.09** | **10.80** | **12.57** | **12.47** | **10.10** | **15.54** | **16.15** | **33.46** | **20.12** | **12.23** | **21.32** | 1.74 | **2.882E-02** | **AKAP7** |
| **6.78** | **11.39** | **13.21** | **7.59** | **13.58** | **8.60** | **20.41** | **16.74** | **29.94** | **17.05** | **10.19** | **21.03** | 2.06 | **4.609E-02** | **AKR1C2** |
| **4.86** | **4.98** | **4.15** | **4.87** | **6.64** | **3.52** | **14.39** | **8.66** | **13.34** | **9.59** | **4.84** | **11.50** | 2.38 | **8.091E-06** | **AMER3** |
| **3.70** | **2.60** | **1.36** | **1.38** | **1.37** | **2.95** | **5.35** | **3.73** | **6.79** | **3.44** | **2.23** | **4.83** | 2.17 | **1.367E-05** | **ANK1** |
| **7.64** | **5.85** | **6.36** | **4.81** | **9.34** | **6.39** | **19.89** | **7.57** | **13.66** | **11.66** | **6.73** | **13.19** | 1.96 | **2.325E-02** | **ANKRD34A** |
| **5.62** | **6.90** | **7.05** | **13.37** | **15.30** | **5.29** | **22.13** | **17.26** | **13.88** | **9.47** | **8.92** | **15.69** | 1.76 | **4.879E-02** | **ANKRD44** |
| **22.81** | **33.97** | **18.05** | **20.81** | **35.47** | **21.43** | **43.54** | **40.73** | **59.89** | **43.78** | **25.42** | **46.99** | 1.85 | **2.828E-02** | **AP3B2** |
| **13.71** | **12.98** | **6.96** | **14.13** | **9.95** | **10.02** | **18.10** | **19.25** | **24.63** | **16.89** | **11.29** | **19.72** | 1.75 | **6.758E-03** | **AP4B1** |
| **2.65** | **3.91** | **4.90** | **7.70** | **8.17** | **4.86** | **19.22** | **12.17** | **7.75** | **8.52** | **5.37** | **11.92** | 2.22 | **5.246E-04** | **ARHGAP20** |
| **41.66** | **9.80** | **18.16** | **27.13** | **35.87** | **17.17** | **101.26** | **34.44** | **50.72** | **22.52** | **24.97** | **52.24** | 2.09 | **4.286E-03** | **ARPP21** |
| **4.42** | **4.46** | **4.75** | **6.66** | **7.65** | **4.99** | **8.18** | **9.56** | **10.24** | **10.40** | **5.49** | **9.60** | 1.75 | **5.658E-03** | **ASXL3** |
| **160.59** | **177.58** | **178.74** | **209.04** | **345.27** | **110.59** | **457.53** | **309.22** | **423.28** | **298.22** | **196.97** | **372.06** | 1.89 | **3.619E-02** | **ATP1A3** |
| **61.80** | **66.33** | **69.66** | **119.39** | **60.93** | **36.41** | **193.62** | **240.66** | **109.01** | **135.93** | **69.09** | **169.80** | 2.46 | **2.901E-03** | **AUTS2** |
| **35.67** | **17.26** | **6.93** | **14.27** | **14.47** | **16.08** | **32.12** | **30.33** | **48.65** | **24.92** | **17.45** | **34.00** | 1.95 | **6.132E-05** | **BICDL1** |
| **0.91** | **1.02** | **0.68** | **1.71** | **0.97** | **1.33** | **2.10** | **2.14** | **2.73** | **3.16** | **1.10** | **2.53** | 2.30 | **7.247E-03** | **BMP8A** |
| **17.34** | **21.69** | **26.89** | **32.50** | **37.53** | **19.69** | **75.50** | **38.48** | **50.83** | **48.28** | **25.94** | **53.27** | 2.05 | **9.356E-04** | **BRINP1** |
| **8.68** | **8.32** | **5.93** | **9.14** | **13.62** | **7.53** | **20.31** | **13.74** | **22.21** | **11.87** | **8.87** | **17.03** | 1.92 | **2.405E-02** | **BSN** |
| **0.38** | **0.42** | **0.77** | **0.34** | **1.01** | **1.12** | **1.88** | **0.94** | **1.74** | **3.54** | **0.67** | **2.03** | 3.01 | **2.267E-03** | **BTNL9** |
| **1.01** | **0.84** | **1.29** | **0.88** | **2.07** | **4.39** | **6.75** | **11.18** | **3.42** | **9.54** | **1.75** | **7.72** | 4.42 | **5.194E-09** | **C1QL3** |
| **7.64** | **2.04** | **4.72** | **5.31** | **8.00** | **2.23** | **42.51** | **8.15** | **4.55** | **11.84** | **4.99** | **16.76** | 3.36 | **1.332E-02** | **CABP7** |
| **5.12** | **7.73** | **8.63** | **8.47** | **11.56** | **12.02** | **22.55** | **17.72** | **25.36** | **11.52** | **8.92** | **19.29** | 2.16 | **1.182E-02** | **CACNA1A** |
| **2.72** | **3.60** | **1.59** | **1.68** | **2.32** | **3.56** | **5.51** | **3.09** | **9.06** | **3.85** | **2.58** | **5.38** | 2.09 | **2.414E-02** | **CACNA1I** |
| **24.00** | **23.27** | **15.93** | **28.14** | **32.04** | **17.86** | **28.50** | **43.96** | **51.43** | **55.40** | **23.54** | **44.82** | 1.90 | **3.166E-02** | **CACNA2D1** |
| **30.09** | **36.41** | **13.74** | **9.17** | **9.89** | **30.35** | **26.81** | **13.54** | **61.93** | **53.47** | **21.61** | **38.94** | 1.80 | **9.091E-04** | **CACNA2D2** |
| **4.57** | **4.36** | **2.98** | **2.88** | **3.12** | **3.10** | **5.05** | **5.81** | **9.98** | **10.43** | **3.50** | **7.82** | 2.23 | **1.107E-06** | **CACNG2** |
| **3.81** | **5.37** | **4.54** | **0.55** | **3.90** | **3.80** | **8.71** | **4.49** | **12.89** | **21.67** | **3.66** | **11.94** | 3.26 | **1.881E-02** | **CACNG3** |
| **1.19** | **2.06** | **0.71** | **1.00** | **1.20** | **1.33** | **1.62** | **2.02** | **3.42** | **4.60** | **1.25** | **2.91** | 2.33 | **5.086E-03** | **CADM3-AS1** |
| **20.78** | **39.96** | **30.87** | **36.25** | **55.17** | **26.93** | **68.46** | **58.98** | **86.90** | **57.17** | **34.99** | **67.88** | 1.94 | **2.267E-02** | **CAMK2B** |
| **4.66** | **3.29** | **4.67** | **7.37** | **9.49** | **4.28** | **12.41** | **10.35** | **7.71** | **8.45** | **5.63** | **9.73** | 1.73 | **2.308E-02** | **CARMIL2** |
| **18.71** | **21.97** | **20.58** | **33.69** | **38.35** | **29.84** | **56.44** | **72.35** | **73.49** | **41.00** | **27.19** | **60.82** | 2.24 | **2.202E-03** | **CARMIL3** |
| **0.94** | **1.24** | **11.75** | **3.40** | **11.83** | **33.33** | **53.15** | **10.00** | **101.69** | **3.96** | **10.41** | **42.20** | 4.05 | **4.237E-02** | **CARTPT** |
| **20.34** | **34.46** | **17.28** | **20.29** | **24.96** | **20.96** | **42.22** | **34.90** | **52.41** | **42.46** | **23.05** | **43.00** | 1.87 | **8.396E-04** | **CASKIN1** |
| **20.96** | **10.82** | **8.84** | **15.76** | **11.19** | **17.15** | **40.97** | **24.80** | **26.39** | **13.30** | **14.12** | **26.37** | 1.87 | **2.414E-02** | **CBFA2T3** |
| **1.47** | **3.20** | **4.55** | **4.66** | **2.94** | **3.74** | **6.12** | **5.56** | **5.39** | **6.58** | **3.42** | **5.91** | 1.73 | **2.220E-02** | **CCDC125** |
| **2.61** | **9.40** | **3.98** | **2.53** | **3.57** | **7.05** | **11.97** | **6.58** | **15.02** | **9.02** | **4.86** | **10.65** | 2.19 | **4.586E-02** | **CCDC85A** |
| **1.64** | **1.86** | **1.31** | **1.79** | **1.61** | **0.96** | **2.52** | **2.44** | **4.74** | **4.40** | **1.53** | **3.52** | 2.30 | **1.221E-02** | **CD8A** |
| **4.99** | **2.72** | **2.03** | **1.45** | **1.48** | **4.49** | **2.59** | **5.01** | **7.93** | **4.75** | **2.86** | **5.07** | 1.77 | **6.336E-03** | **CDH12** |
| **5.48** | **6.93** | **4.31** | **3.09** | **2.11** | **9.49** | **7.55** | **3.18** | **15.59** | **15.89** | **5.23** | **10.55** | 2.02 | **5.412E-03** | **CDH18** |
| **3.25** | **4.00** | **2.76** | **2.16** | **1.97** | **1.86** | **19.48** | **4.59** | **16.83** | **3.90** | **2.67** | **11.20** | 4.20 | **4.772E-03** | **CDH22** |
| **26.72** | **36.50** | **25.90** | **39.57** | **31.29** | **25.72** | **66.82** | **66.07** | **56.82** | **39.53** | **30.95** | **57.31** | 1.85 | **1.124E-03** | **CDH24** |
| **10.85** | **3.02** | **10.63** | **17.72** | **11.82** | **7.49** | **56.64** | **27.75** | **13.59** | **12.98** | **10.26** | **27.74** | 2.71 | **9.710E-04** | **CDH7** |
| **10.75** | **15.53** | **15.04** | **21.15** | **24.29** | **11.51** | **44.23** | **33.07** | **33.58** | **19.23** | **16.38** | **32.53** | 1.99 | **1.437E-03** | **CECR6** |
| **28.20** | **58.72** | **52.97** | **49.31** | **60.33** | **39.38** | **91.05** | **83.39** | **115.98** | **98.61** | **48.15** | **97.26** | 2.02 | **6.799E-05** | **CELF4** |
| **69.39** | **75.35** | **69.26** | **103.20** | **153.09** | **58.62** | **194.79** | **188.81** | **199.28** | **110.19** | **88.15** | **173.27** | 1.97 | **1.265E-02** | **CELF5** |
| **17.88** | **24.23** | **20.09** | **24.72** | **34.13** | **19.48** | **61.28** | **41.17** | **47.25** | **37.85** | **23.42** | **46.89** | 2.00 | **6.192E-04** | **CELF6** |
| **9.32** | **13.40** | **4.37** | **4.44** | **6.17** | **14.62** | **13.33** | **13.34** | **32.21** | **24.19** | **8.72** | **20.77** | 2.38 | **4.895E-06** | **CHD5** |
| **0.74** | **0.40** | **0.27** | **0.43** | **1.60** | **1.18** | **5.29** | **2.69** | **2.30** | **2.33** | **0.77** | **3.15** | 4.09 | **4.637E-04** | **CHRM2** |
| **6.89** | **9.35** | **10.45** | **13.96** | **19.51** | **8.17** | **45.52** | **22.84** | **28.56** | **11.75** | **11.39** | **27.17** | 2.39 | **1.246E-02** | **CHRNA4** |
| **9.69** | **10.60** | **9.96** | **15.63** | **21.89** | **5.85** | **41.63** | **21.03** | **23.82** | **13.04** | **12.27** | **24.88** | 2.03 | **1.830E-02** | **CHRNB2** |
| **11.48** | **18.54** | **10.67** | **7.70** | **21.43** | **14.43** | **29.29** | **21.24** | **32.05** | **30.33** | **14.04** | **28.23** | 2.01 | **4.609E-02** | **CHST1** |
| **5.59** | **6.50** | **7.99** | **5.60** | **8.44** | **11.10** | **32.79** | **13.25** | **13.99** | **14.64** | **7.53** | **18.67** | 2.48 | **1.707E-03** | **CHST8** |
| **14.03** | **20.05** | **6.32** | **12.56** | **14.68** | **22.88** | **22.39** | **22.46** | **44.21** | **32.50** | **15.09** | **30.39** | 2.01 | **1.576E-02** | **CKMT1B** |
| **23.04** | **0.86** | **13.40** | **1.22** | **9.65** | **67.26** | **10.24** | **6.85** | **3.43** | **12.93** | **19.24** | **8.36** | 0.43 | **4.559E-02** | **CLIC6** |
| **17.00** | **3.15** | **6.67** | **12.22** | **12.91** | **6.58** | **42.90** | **22.92** | **21.41** | **14.96** | **9.76** | **25.55** | 2.62 | **1.131E-05** | **CLMP** |
| **8.67** | **6.17** | **11.74** | **15.42** | **17.11** | **6.44** | **27.86** | **23.50** | **28.28** | **14.75** | **10.93** | **23.60** | 2.16 | **5.658E-03** | **CLVS1** |
| **3.38** | **3.19** | **1.54** | **2.41** | **3.66** | **2.13** | **4.79** | **5.56** | **7.93** | **5.42** | **2.72** | **5.92** | 2.18 | **1.676E-02** | **CNKSR1** |
| **3.94** | **2.02** | **3.06** | **3.78** | **3.18** | **2.64** | **6.68** | **5.58** | **4.93** | **4.31** | **3.10** | **5.38** | 1.73 | **4.409E-03** | **CNNM1** |
| **52.00** | **14.32** | **9.06** | **18.81** | **17.40** | **21.78** | **46.33** | **26.08** | **47.39** | **34.14** | **22.23** | **38.48** | 1.73 | **1.325E-04** | **CNTN2** |
| **56.88** | **63.72** | **172.30** | **126.74** | **119.25** | **198.29** | **271.94** | **295.73** | **147.60** | **240.91** | **122.86** | **239.05** | 1.95 | **1.115E-07** | **CNTNAP2** |
| **1.74** | **10.15** | **3.48** | **1.33** | **0.88** | **2.13** | **3.74** | **3.72** | **14.90** | **31.79** | **3.29** | **13.54** | 4.12 | **7.088E-06** | **CNTNAP4** |
| **3.30** | **1.17** | **0.55** | **0.49** | **0.89** | **4.04** | **1.31** | **3.08** | **5.04** | **3.28** | **1.74** | **3.18** | 1.83 | **2.084E-03** | **CNTNAP5** |
| **8.92** | **12.51** | **2.53** | **6.38** | **3.26** | **9.32** | **4.11** | **7.46** | **28.18** | **22.34** | **7.15** | **15.52** | 2.17 | **6.725E-03** | **COL7A1** |
| **345.94** | **20.43** | **29.61** | **16.07** | **32.93** | **246.73** | **45.95** | **26.81** | **33.02** | **74.84** | **115.29** | **45.15** | 0.39 | **7.709E-03** | **COL9A2** |
| **13.77** | **12.85** | **24.51** | **22.80** | **37.95** | **15.84** | **60.04** | **44.46** | **37.04** | **27.54** | **21.29** | **42.27** | 1.99 | **7.289E-04** | **CORO1A** |
| **41.69** | **25.89** | **16.25** | **17.21** | **19.76** | **25.10** | **60.81** | **37.40** | **54.83** | **40.86** | **24.32** | **48.48** | 1.99 | **6.153E-14** | **CPNE5** |
| **2.05** | **9.74** | **1.62** | **2.04** | **1.49** | **12.84** | **74.19** | **24.28** | **86.97** | **0.84** | **4.97** | **46.57** | 9.38 | **4.865E-02** | **CPNE6** |
| **2.55** | **3.71** | **2.29** | **4.05** | **2.38** | **3.60** | **4.00** | **11.96** | **10.78** | **14.69** | **3.09** | **10.36** | 3.35 | **1.371E-05** | **CPNE7** |
| **10.17** | **5.68** | **8.62** | **7.13** | **7.97** | **12.40** | **22.30** | **6.56** | **14.02** | **30.04** | **8.66** | **18.23** | 2.10 | **2.163E-03** | **CUX2** |
| **1.63** | **0.70** | **0.59** | **3.26** | **0.92** | **1.26** | **7.28** | **5.61** | **3.38** | **2.18** | **1.39** | **4.61** | 3.31 | **4.337E-02** | **DCLK3** |
| **0.79** | **1.09** | **0.95** | **0.71** | **0.41** | **1.38** | **1.88** | **1.79** | **2.52** | **2.25** | **0.89** | **2.11** | 2.37 | **1.518E-02** | **DCTN1-AS1** |
| **17.27** | **6.19** | **50.79** | **33.33** | **57.36** | **21.70** | **115.66** | **38.14** | **36.55** | **71.02** | **31.11** | **65.34** | 2.10 | **5.519E-03** | **DDN** |
| **24.74** | **17.92** | **40.26** | **24.45** | **39.56** | **26.95** | **71.69** | **36.92** | **64.28** | **44.51** | **28.98** | **54.35** | 1.88 | **2.101E-02** | **DIRAS2** |
| **10.70** | **18.70** | **17.63** | **21.40** | **31.20** | **13.81** | **66.35** | **32.43** | **36.53** | **25.05** | **18.91** | **40.09** | 2.12 | **2.056E-02** | **DISP2** |
| **16.40** | **12.14** | **17.95** | **38.87** | **23.72** | **8.79** | **49.54** | **55.19** | **35.60** | **28.22** | **19.65** | **42.14** | 2.14 | **1.081E-02** | **DISP3** |
| **9.99** | **11.41** | **12.80** | **9.60** | **20.12** | **9.65** | **27.60** | **21.97** | **31.49** | **21.34** | **12.26** | **25.60** | 2.09 | **9.363E-03** | **DLGAP3** |
| **52.66** | **33.31** | **60.06** | **69.03** | **106.89** | **55.72** | **158.35** | **140.13** | **104.60** | **61.96** | **62.95** | **116.26** | 1.85 | **4.706E-03** | **DMTN** |
| **11.01** | **12.05** | **18.01** | **22.08** | **28.02** | **12.86** | **36.85** | **40.51** | **22.28** | **27.29** | **17.34** | **31.73** | 1.83 | **4.374E-04** | **DOC2A** |
| **35.27** | **32.87** | **43.19** | **55.23** | **76.12** | **31.68** | **161.56** | **111.81** | **99.89** | **47.06** | **45.73** | **105.08** | 2.30 | **7.933E-05** | **DPF1** |
| **7.19** | **6.70** | **2.61** | **1.34** | **1.97** | **4.57** | **2.07** | **3.93** | **16.36** | **12.97** | **4.06** | **8.83** | 2.17 | **1.392E-03** | **DRP2** |
| **3.22** | **4.38** | **4.17** | **5.97** | **3.90** | **3.04** | **6.93** | **11.01** | **8.85** | **9.37** | **4.11** | **9.04** | 2.20 | **8.269E-06** | **DSCAM** |
| **10.50** | **17.30** | **24.21** | **25.40** | **30.78** | **14.94** | **32.87** | **40.43** | **47.07** | **63.79** | **20.52** | **46.04** | 2.24 | **1.435E-04** | **ELAVL2** |
| **5.61** | **11.12** | **3.12** | **6.28** | **3.90** | **4.88** | **7.81** | **8.18** | **14.14** | **12.42** | **5.82** | **10.64** | 1.83 | **3.215E-02** | **EME2** |
| **1.57** | **2.38** | **0.83** | **1.31** | **1.26** | **1.20** | **1.89** | **1.76** | **3.03** | **4.92** | **1.43** | **2.90** | 2.04 | **1.742E-02** | **EML5** |
| **7.72** | **6.30** | **3.19** | **5.60** | **8.19** | **8.38** | **14.95** | **11.54** | **14.40** | **11.95** | **6.56** | **13.21** | 2.01 | **3.250E-03** | **EPHA10** |
| **15.06** | **8.44** | **27.48** | **39.04** | **48.55** | **13.16** | **53.76** | **66.22** | **32.25** | **39.29** | **25.29** | **47.88** | 1.89 | **1.490E-02** | **EPHA5** |
| **2.37** | **5.13** | **4.08** | **3.58** | **3.00** | **2.40** | **9.15** | **5.98** | **13.93** | **13.91** | **3.43** | **10.74** | 3.13 | **1.010E-05** | **EPHA8** |
| **16.92** | **8.57** | **5.96** | **11.07** | **11.01** | **7.35** | **16.64** | **21.22** | **23.10** | **12.19** | **10.15** | **18.29** | 1.80 | **3.852E-02** | **FAAH** |
| **3.55** | **7.24** | **2.60** | **3.25** | **5.27** | **3.08** | **8.97** | **10.60** | **12.33** | **11.50** | **4.16** | **10.85** | 2.61 | **3.853E-04** | **FAM19A2** |
| **2.81** | **3.56** | **2.56** | **4.44** | **2.64** | **1.46** | **7.24** | **5.30** | **6.52** | **5.20** | **2.91** | **6.06** | 2.08 | **3.100E-02** | **FAM212B-AS1** |
| **25.33** | **21.91** | **20.35** | **19.49** | **10.21** | **21.17** | **48.30** | **26.56** | **57.76** | **24.97** | **19.74** | **39.40** | 2.00 | **2.796E-02** | **FAM65B** |
| **2.33** | **3.31** | **6.25** | **5.87** | **12.04** | **3.65** | **18.79** | **13.23** | **10.03** | **5.63** | **5.58** | **11.92** | 2.14 | **2.419E-02** | **FAM78B** |
| **18.94** | **22.78** | **20.87** | **22.26** | **36.47** | **18.56** | **40.49** | **38.25** | **47.66** | **32.96** | **23.31** | **39.84** | 1.71 | **4.546E-02** | **FBXO41** |
| **16.90** | **18.61** | **20.71** | **23.47** | **28.36** | **18.26** | **41.56** | **46.64** | **39.86** | **31.24** | **21.05** | **39.83** | 1.89 | **9.834E-06** | **FCHO1** |
| **5.78** | **17.51** | **6.90** | **5.65** | **6.04** | **8.28** | **14.66** | **15.69** | **17.33** | **19.29** | **8.36** | **16.74** | 2.00 | **3.038E-03** | **FGF12** |
| **2.56** | **5.10** | **0.65** | **1.83** | **1.02** | **2.07** | **2.23** | **3.22** | **10.48** | **4.69** | **2.20** | **5.16** | 2.34 | **2.315E-02** | **FGF17** |
| **6.67** | **4.64** | **6.00** | **7.91** | **10.31** | **5.62** | **13.33** | **13.48** | **11.49** | **9.80** | **6.86** | **12.02** | 1.75 | **5.658E-03** | **FMNL1** |
| **1.22** | **1.36** | **1.53** | **1.16** | **1.35** | **1.29** | **3.03** | **1.84** | **2.93** | **3.67** | **1.32** | **2.87** | 2.18 | **2.245E-02** | **FOXH1** |
| **6.23** | **11.37** | **16.98** | **5.95** | **6.75** | **24.89** | **36.24** | **12.46** | **32.50** | **50.87** | **12.03** | **33.02** | 2.74 | **1.110E-04** | **FSTL5** |
| **1.32** | **1.36** | **1.23** | **0.85** | **0.92** | **1.84** | **2.34** | **1.57** | **3.10** | **3.24** | **1.25** | **2.56** | 2.05 | **1.416E-03** | **FUT1** |
| **2.85** | **2.37** | **0.81** | **0.86** | **1.95** | **3.90** | **4.00** | **5.96** | **6.37** | **12.26** | **2.12** | **7.14** | 3.37 | **1.558E-06** | **GABRA1** |
| **26.66** | **33.79** | **40.31** | **38.56** | **53.94** | **28.37** | **92.65** | **64.07** | **76.34** | **39.08** | **36.94** | **68.03** | 1.84 | **3.923E-02** | **GDAP1L1** |
| **0.48** | **0.66** | **1.56** | **1.36** | **1.37** | **6.97** | **2.35** | **1.03** | **0.87** | **25.69** | **2.07** | **7.49** | 3.62 | **3.373E-03** | **GDF7** |
| **61.50** | **86.98** | **71.64** | **103.87** | **127.80** | **39.30** | **189.49** | **142.35** | **208.52** | **92.32** | **81.85** | **158.17** | 1.93 | **2.993E-02** | **GNG4** |
| **8.75** | **9.84** | **11.77** | **9.48** | **23.03** | **8.07** | **34.42** | **23.39** | **25.23** | **15.89** | **11.82** | **24.73** | 2.09 | **2.108E-02** | **GOLGA7B** |
| **0.68** | **1.07** | **0.38** | **0.39** | **0.27** | **1.26** | **3.90** | **0.91** | **4.12** | **1.32** | **0.67** | **2.56** | 3.80 | **4.737E-03** | **GPR179** |
| **0.88** | **2.47** | **1.15** | **0.22** | **1.02** | **2.84** | **3.82** | **1.85** | **7.24** | **3.83** | **1.43** | **4.18** | 2.92 | **1.385E-02** | **GPR26** |
| **30.75** | **34.42** | **52.15** | **58.30** | **86.18** | **30.07** | **146.66** | **95.51** | **96.76** | **54.16** | **48.65** | **98.27** | 2.02 | **2.893E-03** | **GPRIN1** |
| **15.56** | **26.44** | **1.87** | **4.41** | **3.13** | **11.63** | **8.67** | **6.70** | **47.93** | **12.39** | **10.51** | **18.92** | 1.80 | **4.417E-02** | **GRIP2** |
| **2.39** | **2.29** | **13.66** | **5.67** | **5.48** | **11.12** | **4.79** | **7.22** | **14.50** | **28.62** | **6.77** | **13.78** | 2.04 | **1.263E-02** | **GRM1** |
| **10.86** | **9.67** | **24.38** | **11.28** | **7.29** | **36.55** | **8.50** | **16.84** | **9.20** | **83.67** | **16.67** | **29.55** | 1.77 | **4.641E-02** | **GSTM1** |
| **37.19** | **9.09** | **10.28** | **12.29** | **11.28** | **23.67** | **62.32** | **35.68** | **33.16** | **16.57** | **17.30** | **36.93** | 2.13 | **9.788E-06** | **GUCY1A3** |
| **13.86** | **29.99** | **25.66** | **18.75** | **26.14** | **30.66** | **43.64** | **45.47** | **51.90** | **58.97** | **24.18** | **49.99** | 2.07 | **1.462E-03** | **GUSBP9** |
| **4.82** | **5.57** | **2.34** | **3.66** | **2.85** | **4.98** | **6.99** | **5.37** | **10.74** | **6.27** | **4.04** | **7.34** | 1.82 | **3.332E-02** | **HAPLN2** |
| **4.76** | **7.75** | **1.09** | **1.06** | **0.42** | **1.70** | **5.66** | **1.95** | **10.10** | **4.55** | **2.80** | **5.56** | 1.99 | **2.496E-02** | **HAR1A** |
| **9.16** | **6.54** | **5.30** | **5.77** | **6.52** | **8.24** | **38.69** | **12.11** | **18.74** | **9.00** | **6.92** | **19.64** | 2.84 | **8.819E-04** | **HAS3** |
| **9.21** | **9.43** | **8.57** | **8.01** | **13.21** | **8.01** | **22.85** | **13.85** | **24.62** | **18.53** | **9.41** | **19.96** | 2.12 | **1.804E-03** | **HCN2** |
| **0.54** | **0.59** | **0.42** | **0.13** | **0.20** | **2.91** | **6.12** | **5.01** | **6.05** | **2.51** | **0.80** | **4.92** | 6.16 | **4.895E-06** | **HCRTR2** |
| **19.37** | **22.80** | **25.16** | **37.67** | **26.84** | **30.46** | **58.83** | **54.13** | **52.04** | **32.87** | **27.05** | **49.47** | 1.83 | **1.932E-02** | **HIST2H2BE** |
| **326.07** | **362.64** | **383.38** | **448.22** | **668.31** | **262.66** | **967.96** | **733.11** | **982.89** | **574.95** | **408.55** | **814.73** | 1.99 | **6.336E-03** | **HMP19** |
| **19.62** | **36.71** | **27.41** | **15.64** | **21.43** | **27.64** | **66.74** | **29.35** | **81.30** | **52.47** | **24.74** | **57.47** | 2.32 | **6.057E-03** | **HPCAL4** |
| **1.73** | **0.74** | **0.95** | **0.21** | **0.88** | **1.27** | **2.61** | **1.69** | **3.08** | **3.00** | **0.96** | **2.59** | 2.69 | **5.166E-04** | **HPGD** |
| **1.39** | **1.55** | **0.42** | **1.04** | **0.55** | **0.90** | **2.78** | **2.16** | **3.07** | **2.49** | **0.97** | **2.63** | 2.70 | **1.279E-02** | **HPX** |
| **3.12** | **4.66** | **5.59** | **4.48** | **5.77** | **2.25** | **40.80** | **14.78** | **11.75** | **12.55** | **4.31** | **19.97** | 4.63 | **1.419E-05** | **HS3ST2** |
| **0.58** | **1.50** | **0.61** | **0.58** | **0.92** | **1.13** | **1.07** | **3.68** | **2.38** | **3.84** | **0.89** | **2.74** | 3.10 | **7.910E-03** | **HS3ST5** |
| **3.69** | **3.34** | **1.55** | **3.00** | **2.42** | **3.04** | **2.74** | **4.08** | **6.73** | **6.29** | **2.84** | **4.96** | 1.75 | **4.671E-02** | **HTATSF1P2** |
| **6.29** | **14.69** | **7.42** | **14.13** | **11.16** | **7.86** | **19.74** | **25.84** | **22.02** | **14.10** | **10.26** | **20.43** | 1.99 | **1.421E-02** | **IGDCC3** |
| **76.64** | **58.65** | **199.45** | **273.65** | **251.20** | **50.93** | **384.75** | **426.07** | **209.26** | **202.30** | **151.75** | **305.60** | 2.01 | **2.305E-02** | **IGFBPL1** |
| **24.04** | **22.36** | **69.27** | **80.79** | **124.50** | **42.29** | **181.25** | **127.31** | **73.46** | **51.64** | **60.54** | **108.42** | 1.79 | **1.081E-02** | **MARCH4** |
| **6.44** | **10.71** | **3.86** | **4.66** | **6.60** | **5.49** | **8.82** | **10.38** | **15.76** | **14.22** | **6.29** | **12.30** | 1.95 | **4.061E-03** | **KCNA3** |
| **6.14** | **0.81** | **2.68** | **4.69** | **6.94** | **2.11** | **14.27** | **7.67** | **5.34** | **5.14** | **3.89** | **8.11** | 2.08 | **2.400E-02** | **KCNB2** |
| **5.65** | **13.20** | **8.97** | **7.15** | **9.90** | **10.76** | **23.21** | **13.57** | **20.65** | **19.86** | **9.27** | **19.32** | 2.08 | **8.110E-03** | **KCNC4** |
| **13.86** | **13.67** | **18.04** | **9.35** | **10.37** | **9.13** | **30.89** | **11.62** | **29.01** | **37.78** | **12.40** | **27.33** | 2.20 | **3.119E-02** | **KCNF1** |
| **3.58** | **2.38** | **5.24** | **7.02** | **8.88** | **4.18** | **14.15** | **16.71** | **5.80** | **5.46** | **5.21** | **10.53** | 2.02 | **1.472E-02** | **KCNH3** |
| **23.11** | **53.50** | **27.27** | **28.64** | **34.57** | **35.72** | **60.34** | **40.87** | **65.19** | **71.67** | **33.80** | **59.51** | 1.76 | **2.681E-02** | **KCNIP1** |
| **4.69** | **2.20** | **5.11** | **7.60** | **9.70** | **4.29** | **28.81** | **17.83** | **10.83** | **6.28** | **5.60** | **15.94** | 2.85 | **3.410E-05** | **KCNK12** |
| **13.01** | **13.59** | **8.20** | **8.37** | **10.20** | **6.02** | **24.19** | **11.60** | **27.29** | **15.98** | **9.90** | **19.77** | 2.00 | **8.225E-03** | **KCNK3** |
| **1.11** | **2.02** | **7.48** | **9.21** | **11.45** | **3.65** | **18.49** | **8.43** | **16.15** | **9.46** | **5.82** | **13.13** | 2.26 | **4.319E-02** | **KCNK9** |
| **0.78** | **1.99** | **1.33** | **1.33** | **1.29** | **0.38** | **1.25** | **4.97** | **3.30** | **4.89** | **1.18** | **3.60** | 3.05 | **3.606E-02** | **KCNMB2** |
| **8.67** | **7.91** | **17.73** | **23.01** | **26.18** | **7.43** | **35.84** | **42.11** | **19.78** | **17.55** | **15.16** | **28.82** | 1.90 | **6.016E-03** | **KCNN1** |
| **4.34** | **3.57** | **2.18** | **1.41** | **2.95** | **9.96** | **6.52** | **3.60** | **7.55** | **11.79** | **4.07** | **7.37** | 1.81 | **3.740E-04** | **KIAA1024** |
| **5.24** | **3.83** | **2.30** | **3.03** | **2.68** | **6.73** | **2.90** | **5.14** | **6.65** | **12.50** | **3.97** | **6.80** | 1.71 | **8.303E-03** | **KLF3-AS1** |
| **0.69** | **6.37** | **3.37** | **1.10** | **0.88** | **2.69** | **5.15** | **3.27** | **10.57** | **16.47** | **2.52** | **8.86** | 3.52 | **6.576E-04** | **KLHL1** |
| **129.78** | **2.68** | **21.16** | **2.54** | **13.25** | **131.41** | **54.20** | **9.22** | **6.26** | **26.82** | **50.14** | **24.12** | 0.48 | **1.480E-02** | **KRT8** |
| **8.86** | **5.30** | **1.97** | **1.39** | **1.78** | **3.84** | **4.39** | **2.41** | **17.25** | **11.07** | **3.86** | **8.78** | 2.28 | **7.957E-05** | **LGI2** |
| **1.57** | **2.27** | **0.97** | **2.12** | **1.27** | **2.45** | **3.24** | **3.60** | **5.08** | **3.71** | **1.77** | **3.91** | 2.20 | **1.441E-02** | **LINC00176** |
| **43.05** | **58.72** | **14.08** | **52.47** | **22.22** | **22.70** | **94.82** | **112.29** | **132.50** | **49.88** | **35.54** | **97.37** | 2.74 | **1.375E-03** | **LINC00599** |
| **3.02** | **2.70** | **1.51** | **1.46** | **0.97** | **3.23** | **4.44** | **2.93** | **6.71** | **3.69** | **2.15** | **4.44** | 2.07 | **1.705E-02** | **LINC01783** |
| **5.30** | **2.26** | **1.89** | **2.48** | **2.35** | **2.81** | **9.33** | **4.58** | **7.55** | **2.64** | **2.85** | **6.03** | 2.12 | **2.028E-02** | **LINC01801** |
| **72.70** | **57.14** | **127.11** | **167.65** | **250.79** | **103.62** | **401.66** | **265.58** | **173.27** | **110.67** | **129.83** | **237.79** | 1.83 | **1.634E-02** | **LINGO1** |
| **103.77** | **28.58** | **48.30** | **186.52** | **255.10** | **102.18** | **823.62** | **621.33** | **288.31** | **88.29** | **120.74** | **455.39** | 3.77 | **3.248E-03** | **LMO3** |
| **3.52** | **2.99** | **2.27** | **2.11** | **2.54** | **1.80** | **3.69** | **3.56** | **5.88** | **4.41** | **2.54** | **4.38** | 1.73 | **4.785E-02** | **LOC145783** |
| **9.68** | **7.42** | **4.65** | **8.66** | **6.45** | **9.85** | **8.68** | **15.20** | **19.85** | **15.62** | **7.79** | **14.84** | 1.91 | **5.595E-03** | **LOC440300** |
| **0.64** | **1.09** | **0.56** | **1.14** | **0.22** | **1.64** | **3.19** | **4.57** | **4.81** | **2.14** | **0.88** | **3.68** | 4.16 | **1.099E-02** | **LOC646241** |
| **9.02** | **5.41** | **109.94** | **79.16** | **75.58** | **164.94** | **125.97** | **123.29** | **49.56** | **237.90** | **74.01** | **134.18** | 1.81 | **9.256E-07** | **LPL** |
| **1.69** | **1.31** | **5.01** | **7.29** | **4.48** | **1.54** | **10.04** | **17.55** | **5.70** | **3.81** | **3.55** | **9.28** | 2.61 | **4.040E-02** | **LRTM2** |
| **10.05** | **11.28** | **12.29** | **11.06** | **17.55** | **14.40** | **38.59** | **13.18** | **24.69** | **27.05** | **12.77** | **25.88** | 2.03 | **1.406E-02** | **LSM11** |
| **4.21** | **2.66** | **2.37** | **2.47** | **2.95** | **2.26** | **6.59** | **5.27** | **5.01** | **3.57** | **2.82** | **5.11** | 1.81 | **6.171E-04** | **MAP10** |
| **44.86** | **54.30** | **42.07** | **55.12** | **73.20** | **46.65** | **80.52** | **92.64** | **105.57** | **86.11** | **52.70** | **91.21** | 1.73 | **9.363E-03** | **MAST1** |
| **36.70** | **33.51** | **21.75** | **40.94** | **34.48** | **24.80** | **75.41** | **61.03** | **67.08** | **35.55** | **32.03** | **59.77** | 1.87 | **1.147E-03** | **MCF2L** |
| **97.87** | **201.55** | **31.90** | **95.03** | **59.82** | **51.89** | **67.40** | **174.12** | **249.42** | **198.43** | **89.68** | **172.35** | 1.92 | **4.591E-02** | **MIAT** |
| **8.78** | **9.60** | **2.08** | **4.50** | **3.08** | **4.47** | **11.49** | **11.37** | **30.60** | **18.10** | **5.42** | **17.89** | 3.30 | **1.020E-20** | **MIR124-2HG** |
| **32.73** | **49.12** | **43.17** | **41.89** | **55.89** | **41.96** | **82.85** | **54.20** | **99.88** | **82.54** | **44.13** | **79.87** | 1.81 | **1.203E-02** | **NAPB** |
| **1.30** | **2.84** | **3.15** | **1.36** | **1.66** | **4.63** | **5.07** | **3.12** | **4.81** | **8.13** | **2.49** | **5.28** | 2.12 | **1.602E-03** | **NDST3** |
| **14.32** | **37.20** | **15.33** | **15.55** | **9.55** | **11.85** | **27.24** | **44.50** | **42.51** | **73.77** | **17.30** | **47.00** | 2.72 | **3.996E-08** | **NEGR1** |
| **8.49** | **11.77** | **6.38** | **6.54** | **10.12** | **9.18** | **15.28** | **13.22** | **26.76** | **13.60** | **8.75** | **17.21** | 1.97 | **1.895E-02** | **NEURL1** |
| **16.33** | **9.18** | **10.15** | **8.24** | **10.63** | **8.47** | **13.76** | **34.99** | **26.12** | **14.30** | **10.50** | **22.29** | 2.12 | **3.820E-02** | **NKAIN2** |
| **1.36** | **6.01** | **6.10** | **1.17** | **1.44** | **4.33** | **3.44** | **8.25** | **5.16** | **18.19** | **3.40** | **8.76** | 2.58 | **4.672E-02** | **NMU** |
| **5.32** | **9.47** | **2.17** | **5.02** | **4.40** | **7.36** | **12.09** | **9.16** | **17.93** | **9.14** | **5.62** | **12.08** | 2.15 | **3.542E-02** | **NPTN-IT1** |
| **1.94** | **5.30** | **4.92** | **1.02** | **3.19** | **9.25** | **1.74** | **5.61** | **6.80** | **30.62** | **4.27** | **11.19** | 2.62 | **1.037E-02** | **OLFM3** |
| **1.57** | **12.76** | **8.72** | **4.05** | **2.85** | **2.87** | **8.27** | **14.82** | **13.11** | **24.77** | **5.47** | **15.24** | 2.79 | **2.658E-03** | **OMG** |
| **16.88** | **25.60** | **11.86** | **16.19** | **21.85** | **14.26** | **53.51** | **24.81** | **47.15** | **24.88** | **17.77** | **37.59** | 2.11 | **2.192E-02** | **PCDH1** |
| **3.79** | **6.59** | **0.85** | **1.62** | **1.10** | **8.88** | **4.64** | **4.86** | **14.03** | **5.67** | **3.80** | **7.30** | 1.92 | **1.112E-02** | **PCDH11X** |
| **5.21** | **8.61** | **9.45** | **4.94** | **9.76** | **37.93** | **27.58** | **14.21** | **20.96** | **59.07** | **12.65** | **30.45** | 2.41 | **6.569E-06** | **PCDHGC4** |
| **3.66** | **2.53** | **0.83** | **1.74** | **1.13** | **2.00** | **2.14** | **2.94** | **6.04** | **3.73** | **1.98** | **3.71** | 1.87 | **3.119E-02** | **PCF11-AS1** |
| **9.03** | **3.84** | **3.14** | **5.98** | **9.08** | **10.45** | **12.85** | **14.58** | **13.27** | **7.35** | **6.92** | **12.01** | 1.74 | **4.309E-02** | **PCLO** |
| **2.53** | **0.44** | **0.56** | **1.49** | **0.22** | **0.32** | **6.54** | **19.27** | **2.42** | **0.72** | **0.93** | **7.24** | 7.81 | **3.445E-02** | **PDYN** |
| **19.08** | **8.78** | **9.04** | **14.25** | **14.99** | **7.68** | **23.75** | **22.84** | **25.79** | **16.27** | **12.30** | **22.16** | 1.80 | **5.547E-03** | **PLCH2** |
| **2.03** | **0.00** | **42.79** | **2.06** | **14.33** | **323.83** | **15.78** | **53.00** | **4.42** | **16.98** | **64.17** | **22.55** | 0.35 | **2.296E-02** | **PMCH** |
| **9.53** | **13.78** | **6.79** | **9.14** | **10.17** | **9.03** | **16.74** | **14.63** | **17.88** | **18.21** | **9.74** | **16.86** | 1.73 | **2.396E-03** | **PNMAL2** |
| **8.94** | **0.64** | **5.04** | **9.43** | **4.39** | **4.72** | **25.24** | **12.04** | **7.61** | **13.21** | **5.53** | **14.53** | 2.63 | **2.491E-02** | **POU3F1** |
| **32.12** | **21.51** | **23.25** | **25.80** | **40.38** | **25.21** | **64.01** | **40.37** | **48.83** | **42.81** | **28.05** | **49.00** | 1.75 | **6.457E-04** | **PPFIA3** |
| **2.65** | **2.70** | **5.38** | **11.30** | **8.14** | **3.54** | **7.46** | **14.97** | **32.99** | **30.51** | **5.62** | **21.48** | 3.82 | **9.209E-03** | **PPIEL** |
| **3.26** | **3.70** | **6.38** | **7.78** | **7.52** | **3.54** | **22.96** | **14.03** | **10.91** | **7.65** | **5.36** | **13.89** | 2.59 | **9.564E-09** | **PPP1R16B** |
| **19.66** | **23.60** | **21.05** | **14.19** | **39.20** | **22.08** | **47.59** | **36.84** | **62.36** | **41.91** | **23.29** | **47.17** | 2.03 | **3.834E-02** | **PPP2R2C** |
| **11.16** | **3.35** | **4.25** | **7.52** | **9.48** | **3.96** | **34.89** | **10.72** | **10.20** | **7.78** | **6.62** | **15.89** | 2.40 | **3.656E-04** | **PRICKLE1** |
| **3.73** | **8.72** | **2.95** | **6.04** | **4.35** | **5.04** | **11.59** | **9.89** | **11.74** | **12.53** | **5.14** | **11.44** | 2.23 | **2.018E-03** | **PTCH2** |
| **4.54** | **1.05** | **2.63** | **2.10** | **2.98** | **6.55** | **9.27** | **13.13** | **3.87** | **14.07** | **3.31** | **10.08** | 3.05 | **9.084E-09** | **PTER** |
| **19.46** | **27.56** | **6.36** | **16.16** | **6.53** | **13.93** | **18.26** | **22.75** | **34.43** | **35.24** | **15.00** | **27.67** | 1.84 | **1.303E-02** | **PTOV1-AS2** |
| **21.51** | **32.55** | **26.52** | **17.29** | **36.21** | **28.12** | **70.13** | **39.90** | **81.27** | **60.16** | **27.03** | **62.86** | 2.33 | **2.901E-03** | **PTPN5** |
| **6.11** | **14.73** | **20.23** | **14.18** | **23.05** | **22.28** | **15.29** | **24.90** | **28.75** | **52.11** | **16.76** | **30.26** | 1.81 | **7.709E-03** | **RAB3B** |
| **13.74** | **20.97** | **16.24** | **9.65** | **19.79** | **13.85** | **19.44** | **23.76** | **44.93** | **55.74** | **15.71** | **35.97** | 2.29 | **5.689E-03** | **RAB3C** |
| **11.12** | **26.40** | **9.49** | **13.52** | **11.89** | **9.42** | **27.51** | **20.77** | **48.39** | **29.81** | **13.64** | **31.62** | 2.32 | **5.017E-05** | **RALYL** |
| **7.10** | **6.92** | **3.30** | **5.35** | **3.72** | **6.77** | **6.76** | **6.89** | **18.07** | **12.03** | **5.53** | **10.94** | 1.98 | **8.560E-04** | **RAPGEF4** |
| **4.72** | **2.46** | **1.80** | **2.87** | **3.17** | **1.68** | **3.89** | **7.03** | **6.26** | **7.10** | **2.78** | **6.07** | 2.18 | **1.416E-02** | **RAPGEF5** |
| **2.48** | **5.73** | **9.24** | **6.89** | **10.56** | **3.20** | **15.79** | **13.43** | **12.75** | **10.84** | **6.35** | **13.20** | 2.08 | **2.138E-02** | **RASGEF1C** |
| **11.71** | **8.36** | **5.90** | **5.14** | **6.90** | **13.17** | **20.08** | **11.89** | **32.31** | **11.34** | **8.53** | **18.91** | 2.22 | **1.727E-03** | **RASGRF1** |
| **12.03** | **13.61** | **22.98** | **28.30** | **37.67** | **15.95** | **68.81** | **53.17** | **40.10** | **17.94** | **21.76** | **45.00** | 2.07 | **2.315E-02** | **RBFOX3** |
| **5.22** | **5.72** | **3.54** | **5.84** | **5.43** | **3.05** | **16.53** | **9.03** | **13.54** | **4.27** | **4.80** | **10.84** | 2.26 | **9.860E-03** | **RIMKLA** |
| **40.83** | **42.75** | **34.89** | **52.85** | **53.26** | **27.02** | **62.87** | **67.18** | **96.58** | **65.82** | **41.93** | **73.11** | 1.74 | **1.750E-02** | **RNF165** |
| **0.66** | **0.85** | **5.11** | **2.30** | **1.95** | **20.17** | **1.35** | **4.11** | **3.45** | **26.30** | **5.17** | **8.80** | 1.70 | **7.032E-06** | **RSPO1** |
| **243.60** | **399.76** | **502.74** | **421.79** | **648.48** | **331.85** | **816.82** | **677.00** | **825.44** | **622.70** | **424.70** | **735.49** | 1.73 | **3.959E-02** | **RTN1** |
| **8.11** | **2.70** | **10.09** | **14.70** | **13.53** | **5.38** | **28.79** | **23.72** | **14.79** | **7.68** | **9.08** | **18.74** | 2.06 | **4.335E-02** | **RTN4RL1** |
| **0.77** | **1.57** | **0.41** | **0.32** | **0.46** | **3.26** | **2.63** | **0.61** | **6.64** | **3.23** | **1.13** | **3.28** | 2.90 | **2.457E-02** | **RTP5** |
| **5.15** | **5.88** | **7.58** | **4.79** | **11.47** | **5.08** | **16.30** | **9.72** | **15.36** | **15.21** | **6.66** | **14.15** | 2.12 | **2.379E-02** | **RUNDC3B** |
| **157.45** | **140.07** | **210.76** | **235.74** | **333.93** | **108.14** | **405.13** | **335.10** | **338.93** | **280.41** | **197.68** | **339.89** | 1.72 | **4.237E-02** | **SBK1** |
| **90.76** | **24.36** | **20.69** | **4.37** | **25.89** | **55.01** | **131.59** | **22.15** | **163.56** | **107.41** | **36.85** | **106.18** | 2.88 | **1.061E-02** | **SCG5** |
| **6.11** | **9.46** | **4.57** | **8.07** | **7.75** | **5.76** | **9.77** | **13.01** | **14.65** | **10.48** | **6.95** | **11.98** | 1.72 | **2.056E-02** | **SCN8A** |
| **27.88** | **26.79** | **28.14** | **35.50** | **54.56** | **20.16** | **65.03** | **70.99** | **58.99** | **49.97** | **32.17** | **61.24** | 1.90 | **3.871E-03** | **SCRT1** |
| **66.70** | **77.33** | **74.40** | **193.67** | **192.07** | **27.73** | **326.34** | **309.42** | **158.12** | **84.81** | **105.32** | **219.67** | 2.09 | **3.542E-02** | **SEZ6** |
| **18.43** | **10.26** | **9.22** | **7.86** | **13.18** | **12.42** | **20.37** | **19.46** | **26.11** | **20.97** | **11.89** | **21.72** | 1.83 | **5.189E-06** | **SGIP1** |
| **9.65** | **17.97** | **15.98** | **22.32** | **20.72** | **13.89** | **47.35** | **43.65** | **47.12** | **27.98** | **16.76** | **41.52** | **2.48** | **2.369E-07** | **SHANK1** |
| **12.73** | **11.69** | **7.46** | **14.03** | **14.74** | **11.24** | **30.27** | **32.16** | **35.38** | **16.99** | **11.98** | **28.70** | 2.40 | **1.651E-04** | **SHISA7** |
| **5.04** | **1.01** | **1.14** | **1.13** | **0.80** | **7.18** | **19.26** | **7.19** | **6.93** | **2.59** | **2.72** | **8.99** | 3.31 | **7.760E-04** | **SHISA8** |
| **4.46** | **2.33** | **7.78** | **18.23** | **18.65** | **10.88** | **25.42** | **30.78** | **9.32** | **25.43** | **10.39** | **22.74** | 2.19 | **5.956E-03** | **SIAH3** |
| **5.11** | **7.90** | **3.19** | **2.11** | **3.20** | **6.30** | **12.78** | **7.65** | **20.05** | **20.40** | **4.63** | **15.22** | 3.28 | **4.329E-09** | **SLC12A5** |
| **3.61** | **16.22** | **10.88** | **6.79** | **7.32** | **13.93** | **21.15** | **13.50** | **42.30** | **61.96** | **9.79** | **34.73** | 3.55 | **1.001E-06** | **SLC17A6** |
| **8.82** | **14.21** | **26.32** | **16.69** | **25.49** | **11.62** | **38.02** | **24.21** | **38.18** | **32.80** | **17.19** | **33.30** | 1.94 | **4.523E-02** | **SLC1A6** |
| **14.03** | **18.52** | **11.98** | **12.42** | **14.28** | **19.51** | **42.03** | **23.93** | **39.54** | **29.92** | **15.12** | **33.86** | 2.24 | **4.797E-05** | **SLC6A17** |
| **1.71** | **5.18** | **1.72** | **1.01** | **1.52** | **2.06** | **3.52** | **2.39** | **6.81** | **7.30** | **2.20** | **5.00** | 2.27 | **4.833E-03** | **SLC7A14** |
| **15.21** | **12.59** | **14.96** | **21.09** | **30.01** | **13.34** | **44.19** | **37.24** | **41.98** | **23.58** | **17.87** | **36.75** | 2.06 | **1.455E-03** | **SLC8A2** |
| **4.65** | **6.81** | **11.01** | **6.67** | **14.04** | **8.78** | **32.95** | **13.91** | **21.50** | **12.49** | **8.66** | **20.21** | 2.33 | **2.354E-02** | **SLC8A3** |
| **10.29** | **12.29** | **12.98** | **14.25** | **15.34** | **7.43** | **19.80** | **23.72** | **41.89** | **18.76** | **12.10** | **26.04** | 2.15 | **1.521E-02** | **SLITRK1** |
| **52.24** | **66.64** | **66.91** | **77.54** | **78.81** | **57.14** | **281.25** | **190.71** | **117.68** | **83.56** | **66.55** | **168.30** | 2.53 | **4.509E-04** | **SNCB** |
| **9.34** | **2.66** | **3.91** | **0.22** | **2.81** | **5.61** | **6.99** | **1.10** | **12.71** | **36.98** | **4.09** | **14.44** | 3.53 | **2.690E-02** | **SNCG** |
| **2.33** | **0.98** | **1.11** | **0.75** | **1.38** | **2.30** | **5.09** | **1.98** | **3.67** | **2.17** | **1.48** | **3.23** | 2.19 | **2.697E-03** | **SOWAHA** |
| **15.27** | **4.14** | **5.74** | **0.78** | **3.29** | **5.77** | **21.76** | **2.43** | **30.70** | **24.72** | **5.83** | **19.90** | 3.41 | **3.688E-02** | **SPOCK3** |
| **18.17** | **23.56** | **14.90** | **23.77** | **25.56** | **16.17** | **33.59** | **35.33** | **36.25** | **34.90** | **20.35** | **35.02** | 1.72 | **2.800E-03** | **SRCIN1** |
| **13.33** | **12.77** | **11.73** | **14.08** | **17.29** | **16.52** | **29.55** | **23.21** | **26.58** | **20.05** | **14.29** | **24.85** | 1.74 | **6.431E-04** | **SSH2** |
| **1.94** | **3.77** | **5.90** | **1.41** | **3.20** | **6.60** | **2.85** | **14.16** | **11.97** | **24.90** | **3.81** | **13.47** | 3.54 | **4.032E-05** | **SSTR1** |
| **38.98** | **51.82** | **49.42** | **55.09** | **76.07** | **37.02** | **115.74** | **80.20** | **104.27** | **78.13** | **51.40** | **94.59** | 1.84 | **4.409E-03** | **STX1B** |
| **6.61** | **6.43** | **1.16** | **0.64** | **1.52** | **2.52** | **2.69** | **1.22** | **10.99** | **10.07** | **3.15** | **6.25** | 1.98 | **4.321E-02** | **STXBP5-AS1** |
| **25.53** | **24.80** | **28.43** | **44.48** | **52.96** | **28.03** | **61.66** | **77.84** | **64.96** | **38.44** | **34.04** | **60.73** | 1.78 | **4.707E-02** | **SULT4A1** |
| **4.98** | **7.23** | **9.51** | **7.97** | **14.50** | **7.32** | **20.52** | **12.83** | **20.31** | **12.70** | **8.58** | **16.59** | 1.93 | **4.372E-02** | **SVOP** |
| **11.26** | **14.41** | **18.30** | **8.81** | **22.66** | **19.49** | **37.83** | **30.48** | **31.73** | **21.86** | **15.82** | **30.48** | 1.93 | **3.581E-02** | **SYN2** |
| **10.00** | **9.87** | **12.53** | **14.26** | **20.14** | **27.45** | **42.49** | **30.32** | **44.27** | **29.74** | **15.71** | **36.71** | 2.34 | **3.154E-04** | **SYN3** |
| **5.78** | **1.84** | **5.24** | **3.16** | **4.12** | **6.47** | **10.93** | **3.36** | **4.59** | **13.70** | **4.44** | **8.15** | 1.84 | **6.984E-03** | **SYNJ2** |
| **51.87** | **40.73** | **74.28** | **41.27** | **72.74** | **73.20** | **174.35** | **120.41** | **106.73** | **111.37** | **59.02** | **128.22** | 2.17 | **1.635E-07** | **SYT13** |
| **9.30** | **23.88** | **25.28** | **19.35** | **24.04** | **20.75** | **50.87** | **39.93** | **51.07** | **39.98** | **20.43** | **45.46** | 2.22 | **5.949E-04** | **SYT3** |
| **29.39** | **15.96** | **7.59** | **12.58** | **16.47** | **22.75** | **26.56** | **22.94** | **44.87** | **29.00** | **17.46** | **30.84** | 1.77 | **3.749E-03** | **TBC1D3L** |
| **7.36** | **3.98** | **5.38** | **7.25** | **5.00** | **5.07** | **9.03** | **13.64** | **11.28** | **8.20** | **5.67** | **10.54** | 1.86 | **4.162E-02** | **TFR2** |
| **3.86** | **3.26** | **0.27** | **0.47** | **0.69** | **2.48** | **4.76** | **0.53** | **9.60** | **6.36** | **1.84** | **5.31** | 2.89 | **1.204E-02** | **TH** |
| **1.99** | **2.03** | **2.30** | **1.34** | **1.76** | **1.68** | **3.68** | **4.88** | **7.01** | **3.80** | **1.85** | **4.84** | 2.62 | **5.566E-04** | **THCAT155** |
| **29.07** | **40.34** | **14.22** | **21.71** | **33.45** | **27.72** | **68.82** | **51.75** | **97.92** | **73.43** | **27.75** | **72.98** | 2.63 | **7.137E-05** | **TMEM145** |
| **3.43** | **4.65** | **1.53** | **4.12** | **2.14** | **0.55** | **10.05** | **7.68** | **12.91** | **7.40** | **2.74** | **9.51** | 3.47 | **9.991E-06** | **TMEM179** |
| **28.83** | **22.92** | **22.24** | **29.05** | **34.71** | **15.17** | **74.86** | **41.15** | **35.30** | **24.80** | **25.49** | **44.03** | 1.73 | **1.895E-02** | **TMEM198** |
| **1.98** | **3.86** | **3.34** | **1.33** | **2.03** | **2.50** | **4.95** | **3.90** | **5.76** | **7.52** | **2.51** | **5.54** | 2.21 | **1.612E-02** | **TMEM266** |
| **16.59** | **24.69** | **14.68** | **13.72** | **24.36** | **27.51** | **25.98** | **29.92** | **61.04** | **39.87** | **20.26** | **39.20** | 1.93 | **3.716E-02** | **TMEM74B** |
| **19.56** | **22.29** | **11.76** | **18.58** | **24.28** | **15.94** | **40.77** | **38.37** | **39.65** | **26.12** | **18.74** | **36.23** | 1.93 | **3.888E-03** | **TRIM46** |
| **1.60** | **2.60** | **1.41** | **0.72** | **1.75** | **1.39** | **13.89** | **1.68** | **3.51** | **4.44** | **1.58** | **5.88** | 3.73 | **4.766E-02** | **TRIM58** |
| **3.86** | **4.87** | **2.90** | **1.66** | **2.74** | **3.99** | **2.78** | **2.78** | **8.40** | **14.24** | **3.34** | **7.05** | 2.11 | **2.513E-02** | **TRIM7** |
| **1.86** | **1.30** | **2.97** | **2.44** | **2.96** | **3.06** | **2.84** | **3.40** | **3.66** | **6.75** | **2.43** | **4.16** | 1.71 | **3.021E-02** | **TRPC3** |
| **212.48** | **3.98** | **26.51** | **2.11** | **16.45** | **468.51** | **28.82** | **25.71** | **18.98** | **41.72** | **121.67** | **28.81** | 0.24 | **3.254E-02** | **TRPM3** |
| **11.94** | **12.01** | **8.46** | **7.16** | **7.43** | **13.18** | **13.38** | **6.74** | **27.98** | **22.92** | **10.03** | **17.75** | 1.77 | **3.283E-02** | **TUNAR** |
| **11.20** | **21.94** | **18.62** | **18.72** | **25.30** | **5.45** | **48.94** | **31.32** | **52.30** | **23.10** | **16.87** | **38.92** | 2.31 | **2.020E-02** | **UBE2QL1** |
| **7.70** | **3.32** | **4.09** | **5.46** | **3.53** | **2.11** | **13.01** | **10.31** | **9.60** | **5.81** | **4.37** | **9.68** | 2.22 | **3.283E-02** | **VSTM2B** |
| **68.95** | **65.24** | **256.97** | **128.59** | **286.08** | **165.26** | **603.02** | **238.53** | **184.13** | **218.61** | **161.85** | **311.07** | 1.92 | **1.178E-02** | **VSTM2L** |
| **16.19** | **19.09** | **17.73** | **12.16** | **7.58** | **10.92** | **45.29** | **17.07** | **40.96** | **24.93** | **13.95** | **32.06** | 2.30 | **3.768E-02** | **WNT7A** |
| **6.99** | **5.18** | **23.04** | **7.40** | **13.41** | **17.87** | **23.42** | **8.55** | **13.67** | **60.81** | **12.32** | **26.62** | 2.16 | **8.231E-03** | **WSCD2** |
| **6.03** | **4.19** | **6.93** | **9.31** | **11.00** | **5.30** | **40.97** | **11.50** | **14.57** | **8.60** | **7.13** | **18.91** | 2.65 | **5.740E-03** | **XKR7** |
| **12.06** | **4.04** | **4.68** | **4.30** | **10.85** | **7.07** | **13.66** | **13.12** | **10.75** | **29.73** | **7.17** | **16.81** | 2.35 | **4.386E-03** | **ZDHHC8P1** |
| **4.23** | **3.95** | **3.40** | **5.45** | **4.46** | **2.23** | **9.65** | **7.06** | **6.17** | **5.36** | **3.95** | **7.06** | 1.79 | **3.833E-02** | **ZNF112** |
| **9.12** | **11.29** | **7.16** | **11.27** | **7.16** | **7.26** | **19.39** | **12.32** | **17.27** | **13.11** | **8.88** | **15.52** | 1.75 | **1.376E-02** | **ZNF202** |
| **5.20** | **6.45** | **3.35** | **5.26** | **4.69** | **4.91** | **10.35** | **5.58** | **13.54** | **11.53** | **4.98** | **10.25** | 2.06 | **6.336E-03** | **ZNF540** |
| **3.92** | **4.44** | **3.48** | **6.28** | **4.37** | **2.36** | **6.98** | **7.28** | **7.58** | **7.42** | **4.14** | **7.32** | 1.77 | **3.283E-02** | **ZNF594** |
| **4.90** | **7.49** | **4.82** | **4.66** | **7.08** | **7.14** | **12.60** | **7.72** | **18.54** | **18.06** | **6.02** | **14.23** | 2.37 | **7.536E-04** | **ZNF804A** |
| **7.71** | **7.76** | **6.27** | **7.11** | **8.80** | **3.41** | **12.25** | **16.49** | **10.95** | **15.24** | **6.84** | **13.73** | 2.01 | **2.216E-02** | **ZSWIM5** |

**Table 4.** The calcium ion transport activity significantly enriched in ***NRXN1****α****^+/-^*** iPSC- derived neurons among the 530 DEGs

| **Pathway ID** | **Pathway description** | **No. genes** | **FDR** |
| --- | --- | --- | --- |
| GO.0005509 | Calcium ion binding | 39 | 5.810E-06 |
| GO.0006816 | Calcium ion transport | 15 | 8.150E-03 |
| GO.0043269 | Regulation of ion transport | 33 | 8.800E-05 |
| GO.0006811 | Ion transport | 39 | 4.110E-02 |
| GO.0030001 | Metal ion transport | 33 | 9.280E-05 |
| GO.0034220 | Ion transmembrane transport | 31 | 3.980E-02 |
| GO.0098660 | Inorganic ion transmembrane transport | 27 | 8.790E-03 |
| GO.0034765 | Regulation of ion transmembrane transport | 27 | 3.650E-05 |
| GO.0005244 | Voltage-gated ion channel activity | 19 | 5.810E-06 |
| GO.0022843 | Voltage-gated cation channel activity | 16 | 4.690E-05 |
| Go.0005245 | Voltage-gated calcium channel activity | 6 | 4.560E-02 |
| Go.0015267 | Channel activity | 23 | 2.280E-03 |
| Go.0005261 | Cation channel activity | 18 | 5.970E-03 |
| Go.0022836 | Gated channel activity | 25 | 5.810E-06 |
| Go.0005216 | Ion channel activity | 21 | 5.970E-03 |
| Go.0022838 | Substrate-specific channel activity | 22 | 3.200E-03 |
| Go.0022891 | Substrate-specific transmembrane transporter activity | 33 | 1.840E-02 |
| Go.0015075 | Ion transmembrane transporter activity | 31 | 2.470E-02 |
| Go.0022857 | Transmembrane transporter activity | 34 | 2.850E-02 |
| Go.0005215 | Transporter activity | 40 | 4.090E-02 |
| Go.0015085 | Calcium ion transmembrane transporter activity | 10 | 4.210E-02 |
| Go.0046873 | Metal ion transmembrane transporter activity | 24 | 9.420E-04 |

The listed 21 pathways in Biological Processes, Molecular Function and Cellular Component of (GO) groups of the STRING analyses that was linked to calcium ion transport, binding and transporter activity with their FDR and number of genes

**Table 5.** Altered calcium signalling in the NRXN1α^+/-^ neurons are associated with 276 upregulated genes, not with 254 down-regulated genes.

| **#Term ID** | **Term Description** | **No.**  **DEGs** | **Background genes** | **FDR** | **matching DEGs** |
| --- | --- | --- | --- | --- | --- |
| GO:0005245 | voltage-gated calcium channel activity | 6 | 40 | 5.50E-04 | *CACNA1A, CACNA1I, CACNA2D1, CACNA2D2, CACNG2, CACNG3* |
| GO:0006816 | calcium ion transport | 12 | 242 | 3.20E-03 | *CACNA1A, CACNA1I, CACNA2D1, CACNA2D2, CACNG2, CACNG3, CHRNA4, CHRNB2, CORO1A, SLC8A2, SLC8A3, TRPC3* |
| GO:0070588 | calcium ion transmembrane transport | 9 | 189 | 2.06E-02 | *CACNA1A, CACNA1I, CACNA2D1, CACNA2D2, CACNG2, CACNG3, SLC8A2, SLC8A3, TRPC3* |
| hsa04020 | Calcium signaling pathway | 8 | 179 | 4.89E-02 | *ADCY1, CACNA1A, CACNA1I, CAMK2B, CHRM2, GRM1, SLC8A2, SLC8A3* |
| HSA-112308 | Presynaptic depolarization and calcium channel opening | 3 | 12 | 2.37E-02 | *CACNA1A, CACNA2D2, CACNG2* |

**Table 6.** Genes of VGCCs associated with neurodevelopmental and neuropsychiatric disorders

| **Pore-forming VGCC subunit** | **Mutations** | **Method** | **Disease** | **References** |
| --- | --- | --- | --- | --- |
| *CACNA1A* (nonsense) | E101Q, S218L, A420V, A713T, A1511S. | Exome sequencing | Epileptic Encephalopathy | [1,2] |
| *CACNA1A* (SNP) | rs7249246; rs12609735 | Association | ASD | [3] |
| *CACNA1B* (nonsense) | Hg19 chr9:140991040 G/A | Exome sequencing | SCZ | [4] |
| *CACNA1B* |  | GeneAnalytics | SCZ | [5] |
| *CACNA1C* (nonsense)  (splice-acceptor) | Hg19 chr12:2717843 C/T  chr12:2706395 G/T | Exome sequencing | SCZ | [4] |
| *CACNA1C* (SNP) | rs1006737 | Association | BPD | [6] |
| *CACNA1C* (nonsense) | **G406R** | Gene sequencing | Timothy Syndrome with ASD | [7.8] |
| *CACNA1C* (SNP) | rs1024582 | Cross-disorder association | ASD, ADHD, SCZ, MDD and BPD | [9] |
| *CACNA1C* | CNV genomic deletion | Cross-disorder association | ASD, Epilepsy, ID | [10] |
| *CACNA1D* (nonsense) | A59V, **G407R, A749G,** V584I, A769G, S1953L, R1997H | Exome sequencing | ASD, Epilepsy | [11-15] |
| *CACNA1D* (nonsense) | **V401L** | Gene sequencing | ASD, Epilepsy | [16] |
| *CACNA1D* (nonsense) | **Q558H** | Gene sequencing | Epilepsy, DD | [17] |
| *CACNA1F* (nonsense) | **I745T** | Gene sequencing | X-linked retinal disorder with male ASD | [18,19] |
| *CACNA1H* (nonsense) | R212C, 902W, W962C, A1874V | Gene sequencing | ASD | [20] |
| *CACNA1H* (frameshift) | Hg19 Chr16:1257835..1257842 (CAAGCTCA/C) | Exome sequencing | SCZ | [4] |
| *CACNA1H* (frameshift) | Hg19 chr16:1257835..1257842  (CAAGCTCA/C) | Exome sequencing | SCZ | [4] |
| *CACNA1H* |  | GeneAnalytics | SCZ | [5] |
| *CACNA1S* (nonsense)  (splice-donor) | Hg19 chr1:201031077 C/T  Hg19 chr1:201056952 A/G | Exome sequencing | SCZ | [4] |
| **Auxiliary β, α2δ & γ subunits** |  |  |  |  |
| *CACNA2D1* | CNV genomic deletion | CNVs | Epilepsy, ID | [21] |
| *CACNA2D1*(frameshift) | Hg19 chr7:81624215 G/GT | Exome sequencing | SCZ | [4] |
| *CACNA2D2* (nonsense)  (nonsense)  (splice-donor) | Hg19 chr3:50402846 G/C  Hg19 chr3:50418227 C/T  Hg19 chr3:50416861 A/C | Exome sequencing | SCZ | [4] |
| *CACNA2D4* (frameshift) | chr12:1902885..1902886 GC/G | Exome sequencing | SCZ | [4] |
| *CACNB2* (nonsense) | **G167S**, S197F, **F240L** | Gene sequencing | ASD | [22,23] |
| *CACNB2* (SNP) | rs2799573 | Cross-disorder association | ASD, ADHD, SCZ, MDD and BPD | [9] |
| *CACNB4* (nonsense) | Hg19 chr2:152695728 C/A | Exome sequencing | SCZ | [4] |
| *CACNG2* (nonsense) | V143L | Gene sequencing | Nonsyndromic ID | [24] |

ADHD = attention deficit hyperactivity disorder; ASD=autism spectrum disorders; BPD=bipolar disorder; DD= Developmental delay; ID = intellectual disability; MDD=major depressive disorder; SCZ = Schizophrenia

**Table** **7.** NRXN1 deletions and other putative CNVs detected by Illumina 1M SNP array in the iPSC lines*

| **Cell lines** | **Chromosome Position (hg19)** | **SNP** | **bp** | **Copy** | **Genes** |
| --- | --- | --- | --- | --- | --- |
| **NCRM1** | chr6:62697745-62922848 | 23 | 225,104 | 1 | KHDRBS2 |
| **1CC1** | chr8:140943503-141150169 | 80 | 206,667 | 1 | TRAPPC9 |
| **2VC1** | chr7:76111938-76615349 | 52 | 503,412 | 3 | DTX2,POMZP3,UPK3B,DTX2P1-UPK3BP1-PMS2P11, LOC100133091 |
| **3VC2** | chr21:36175450-36238507 | 28 | 63,058 | 1 | RUNX1 |
| **3VCX1** | - | - | - | - | - |
| **4C3** | chr2:206359207-206464465  chr11:29859855-30059736 | 34  35 | 105,259  199,882 | 1  3 | PARD3B  KCNA4,LINC01616 |
| **4CX1** | chr20:14306896-14688519 | 110 | 381,624 | 3 | FLRT3,MACROD2,FLRT3,MACROD2-IT1 |
|  |  |  |  |  |  |
| **ND1C1** | **chr2:50711687-51044633**  chr5:74606860-74745228 | **116**  34 | **332,947**  138,369 | 1  1 | **NRXN1**  HMGCR,COL4A3BP |
| **ND2C11** | **chr2:51120335-51360666** | **71** | **240,332** | **1** | **NRXN1** |
| **ND2CX1** | **chr2:51120335-51360666** | **71** | **240,332** | **1** | **NRXN1** |
| **ND4-1C1** | **chr2:50983186-51471321**  chr12:63946056-64118558  chr17:44165803-44345063  chr1:36957830-37353077  chr11:50057854-50629850 | **131**  18  20  101  48 | **488,136**  172,503  179,261  395,248  571,997 | **1**  3  3  3  3 | **NRXN1**  DPY19L2  KANSL1,KANSL1-AS1  GRIK3  LOC441601,LOC646813 |

* The NRXN1 deletions in the patients were originally identified by clinical genetic diagnosis, which were subsequently confirmed by independent SNP arrays of the iPSC lines in the current study. Other putative CNVs require further validation and standardization.

**Table 8.** A list of primary and secondary antibodies used.

| **Primary antibody** | **Manufacture** | **Dilution** | **Species** | **Lot No.** |
| --- | --- | --- | --- | --- |
| OCT4 | Cell Signaling | 1:200 | Rabbit | 2840S |
| SOX2 | Cell Signaling | 1:200 | Rabbit | 3579S |
| SSEA4 | Cell Signaling | 1:200 | Mouse | 4755P2 |
| TRA-1-60 | Cell Signaling | 1:300 | Mouse | 9656 |
| Ki67 | Abcam | 1:500 | Rabbit | Ab15580 |
| P-Histone H3 | Cell Signaling | 1:500 | Mouse | 9706S |
| NESTIN | Abcam | 1:400 | Mouse | Ab18102 |
| PAX6 | Abcam | 1:400 | Rabbit | Ab5790 |
| MAP2 | Abcam | 1:200 | Rabbit | Ab32454 |
| TUJ1 | Abcam | 1:1000 | Mouse | Ab78078 |
| SYNAPSIN1 | Abcam | 1:1000 | Rabbit | Ab8 |
| DCX | Abcam | 1:1000 | Rabbit | Ab18723 |
| GFAP | Dako | 1:200 | Rabbit | 20028619 |
| CTIP2 | Abcam | 1:400 | Rat | Ab18465 |
| TBR1 | Abcam | 1:400 | Rabbit | Ab183032 |
| AFP | Sigma | 1:300 | Mouse | A8452 |
| ASM | Sigma | 1:300 | Mouse | A2547 |
| **Secondary antibody** |  |  |  |  |
| Alexa Fluor 488 | Cell Signaling | 1:1000 | Goat-anti rabbit | 4412S |
| Alexa Fluor 555 | Cell Signaling | 1:1000 | Goat-anti mouse | 4409S |

**Table 9.** RT-PCR primers

| **Genes** | **Forward primer (5’-3’)** | **Reverse primer (5’-3’)** |
| --- | --- | --- |
| ***GAPDH*** | AGGGCTGCTTTTAACTCTGGT | CCCCACTTGATTTTGGAGGGA |
| ***OCT4*** | ACTTCACTGCACTGTACTCCTC | CACCCTTTGTGTTCCCAATTCC |
| ***SOX2*** | AGACTTCACATGTCCCAGCACT | CGGGTTTTCTCCATGCTGTTTC |
| ***NANONG*** | ATAACCTTGGCTGCCGTCTC | ATAACCTTGGCTGCCGTCTC |
| ***FOXG1*** | CCTGCCCTGTGAGTCTTTAAG | GTTCACTTACAGTCTGGTCCC |
| ***NEUROG2*** | CAACGCTGAGGCACAGTTAG | GCTCCTCCTCCTCTTCTTC |
| ***TUBB3*** | GCTCAGGGGCCTTTGGACATCTCTT | TTTTCACACTCCTTCCGCACCACATC |
| ***PAX6*** | CGGTGAATGGGCGGAGTTAT | CCCTCCCATAAGACCAGGAGA |
| ***NES*** | GCACTTCAAGATGTCCCTC | GGGAAGTTGGGCTCAGGACTG |
| ***DLG4*** | AGTCAGAAATACCGCTACCAAG | CCG TTCACCTGCAACTCATATC |
| ***SLC17A7*** | TCAATAACAGCACGACCCAC | TCCTGG AATCTGAGTGACAAT |
| ***SHANK1*** | GAGAGCAAGCAAGAAAGCCG | CCATCCATTAAGCTTGGGGC |
| ***SHANK3*** | ACTCATCCTTCCGCCAACAG | CCCACAGGTGAGTGTGAGAC |
| ***GRIN1*** | AGGCCGTGAGAGACAACAAG | GCCATTCTCGTGGGACTTGA |
| ***GRIN2A*** | CACCGTCTCACCTTCTGCTT | CAGGCCCCAAAGAAGCCATA |
| ***GRIK1*** | TCCCGATGGCAAATATGGGG | CCATTGGGCTTCCGGTAGAG |
| ***GRIK3*** | CAATGCCGTCCAGTCCATCT | GGATGAGCCCTGTACTGTCG |
| ***GRIA4*** | CGGTTGAGCGAATGGTCTCT | ACTGATGGCTCTGCTGATCG |
| ***SATB2*** | TCTCCCCAAACACACCATCA | GCAGCTCCTCGTCCTTAT |
| ***CTIP2*** | AGCAGGAGAACATTGCAGGTA | GGAAATTCATGAGCGGGGACT |
| ***TBR1*** | CGAGCTTCAAATAACAATGGGC | GAGTCTCAGGGAAAGTGAACG |
| ***BRN2*** | AAAGTAACTGTCAAATGCGCG | GCTGTAGTGGTTAGACGCTG |
| ***MAP2*** | CAGTTTCTGCGCCCAGATTTTA | TCCCAATCAATGCTTCCTCG |
| ***CACNA1A*** | CAGACACCAGCCCCATGAAG | CAGACACCAGCCCCATGAAG |

**Table 10.** “N” numbers for the coverslips and calcium recording performed in this study.

| **Cell Line** | **Coverslips (n)** | **Recording (n)** | **Technical replicates (n)** |
| --- | --- | --- | --- |
| 1CC1 | 6 | 14 | 2 |
| 2VC1 | 1 | 3 | 1 |
| 3VC2 | 4 | 9 | 2 |
| 3VCX1 | 9 | 19 | 2 |
| 4CC3 | 7 | 18 | 2 |
| 4CCX1 | 3 | 8 | 2 |
| ND1C1 | 8 | 9 | 2 |
| ND1C4 | 2 | 4 | 1 |
| ND2C11 | 8 | 11 | 2 |
| ND2CX1 | 2 | 4 | 1 |
| ND4-1C1 | 5 | 10 | 2 |
| ND4-1C2 | 4 | 9 | 1 |

**
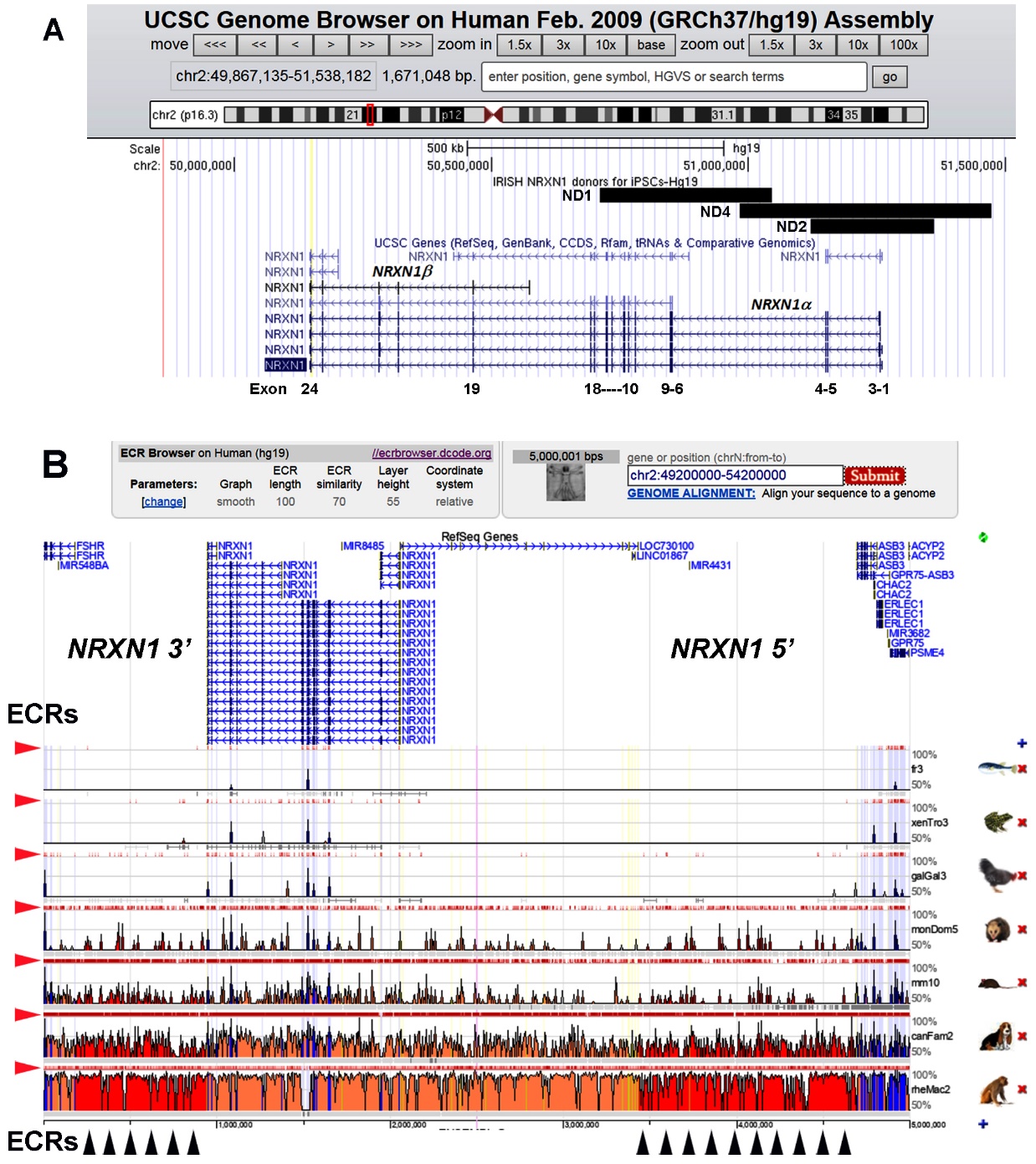
**

**Figure 1. Genome mapping of three ASD patients (ND1, ND2, ND4) and (B) evolutional conservation of the *NRXN1* gene regulation**. (A) The human *NRXN1* gene is located to chr2:50,145,643-51,259,674 on UCSC Genome Browser GRCh37/hg19 Assembly, with 3’ on the short arm and 5’ on the long arm side. The exons are numbers 1-24. Note the exons 19-24 are shared by both NRXN1α and NRXN1β isoforms. Three participants carry deletions in the NRXN1α gene only. The ND1 carries an internal deletion involving exons 6-15, and ND2 and ND4 carry deletions of NRXN1 exons 1-5. Analyses of Evolutional Conservation Regions (ECR) of the NRXN1 gene and franking sequence (5’ and 3’) suggested that the NRXN1 gene is highly regulated by 5MB genomic DNA sequences. The ECRs are colored in Red in the upstream, downstream as well as intron sequences (black arrowheads) and crossed the different species (red arrowheads).


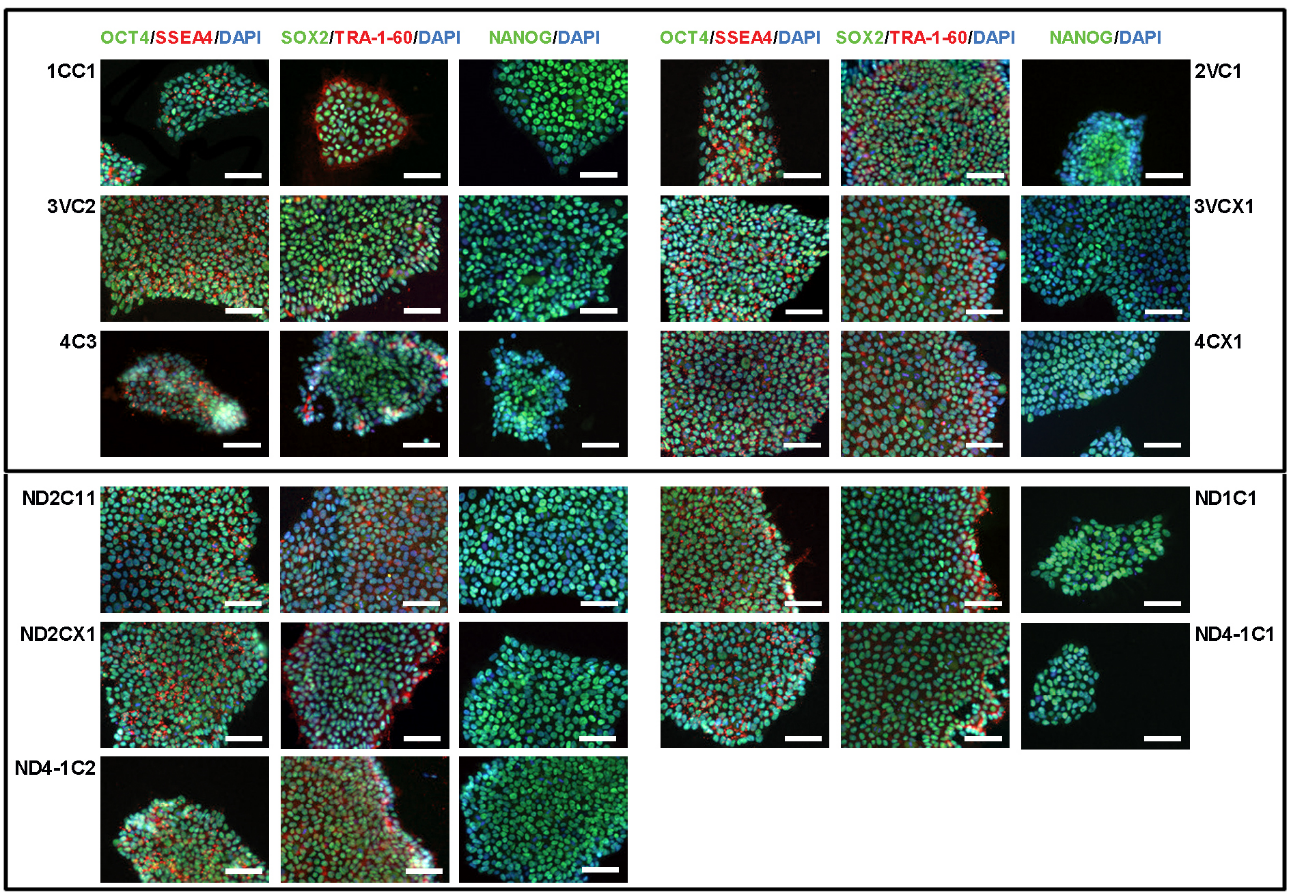


**Figure 2. Characterization of the control and NRXN1α deletion iPSCs by immunocytochemistry.** The pluripotency of iPSC lines were characterized by immunocytochemical staining with OCT4/SSEA4, SOX2/TRA-1-60 and NANOG. DAPI is used to stain all nuclei for quantification. Scale bar 60 μM.

|  |
| --- |

**Figure 3. Neuronal differentiation and early neuronal progenitors.** (A) The dual SMAD inhibition differentiation protocol. (B) Diminishing *OCT4* and increasing *PAX6* mRNA expression during the first 10 days of neural induction. (C) Expression of *PAX6, NES, FOXG1, NEUROG2* mRNA in relation to *GAPDH* at day 20 in control (black) and *NRXN1α*^+/-^ (red) cells. Results shown from 2 independent cortical neuronal differentiation. (D-F) ICC staining of day 20 cultures with neural stem cell markers PAX6 and Nestin.

**Figure 4. Differentiation capability of 100 days iPSC-derived neurons.** (A) mRNA levels of *MAP2* and *SYN1* was measured using qRT-PCR and normalized to *GAPDH*. (B) Representative images of day100 neurons immunostained for MAP2 (red), GFAP (green) and DAPI (blue). (C) The relative cell density was calculated for MAP2 (Control 38±1.53; *NRXN1α^+/-^* 28.67±1.20mm/mm^2^) and GFAP (Control 15±4.73; *NRXN1α^+/-^* 26.67±5.84 mm/mm^2^). (D) Quantitative expression of SYN1 proteins using western blotting showed no significant increase in *NRXN1α* deletion neurons (normalized to GAPDH). All data summary are Mean ± SEM. Scale bar is 25 μM.


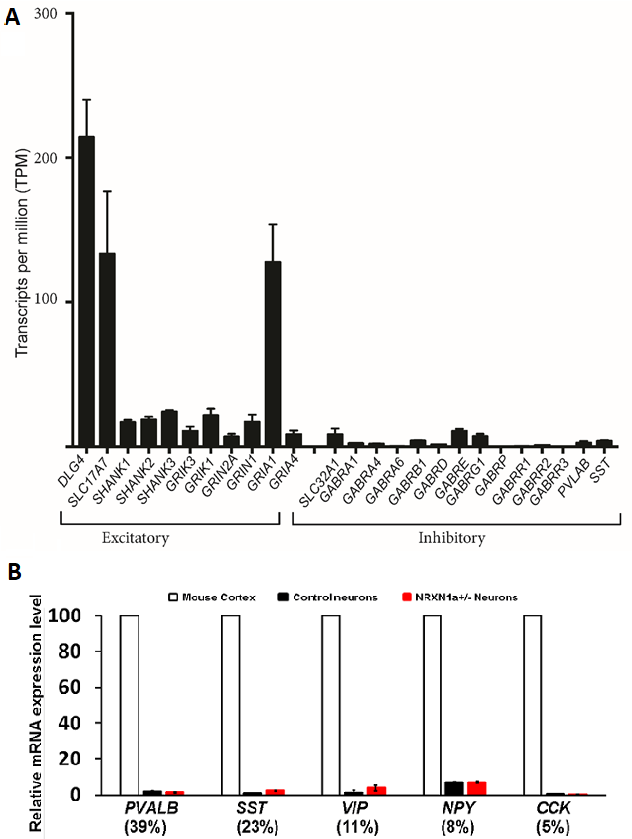


**Figure 5. The iPSC-derived day-100 cortical neurons were largely excitatory.** (A) Transcript per million (TPM) expression using RNA sequencing confirmed the expression of excitatory markers and low expression of GABA transporter (VGAT1) and a series of ionotropic inhibitory GABAergic receptors. RNA was extracted from independent batches of differentiation. All data summary were Mean ± SEM.


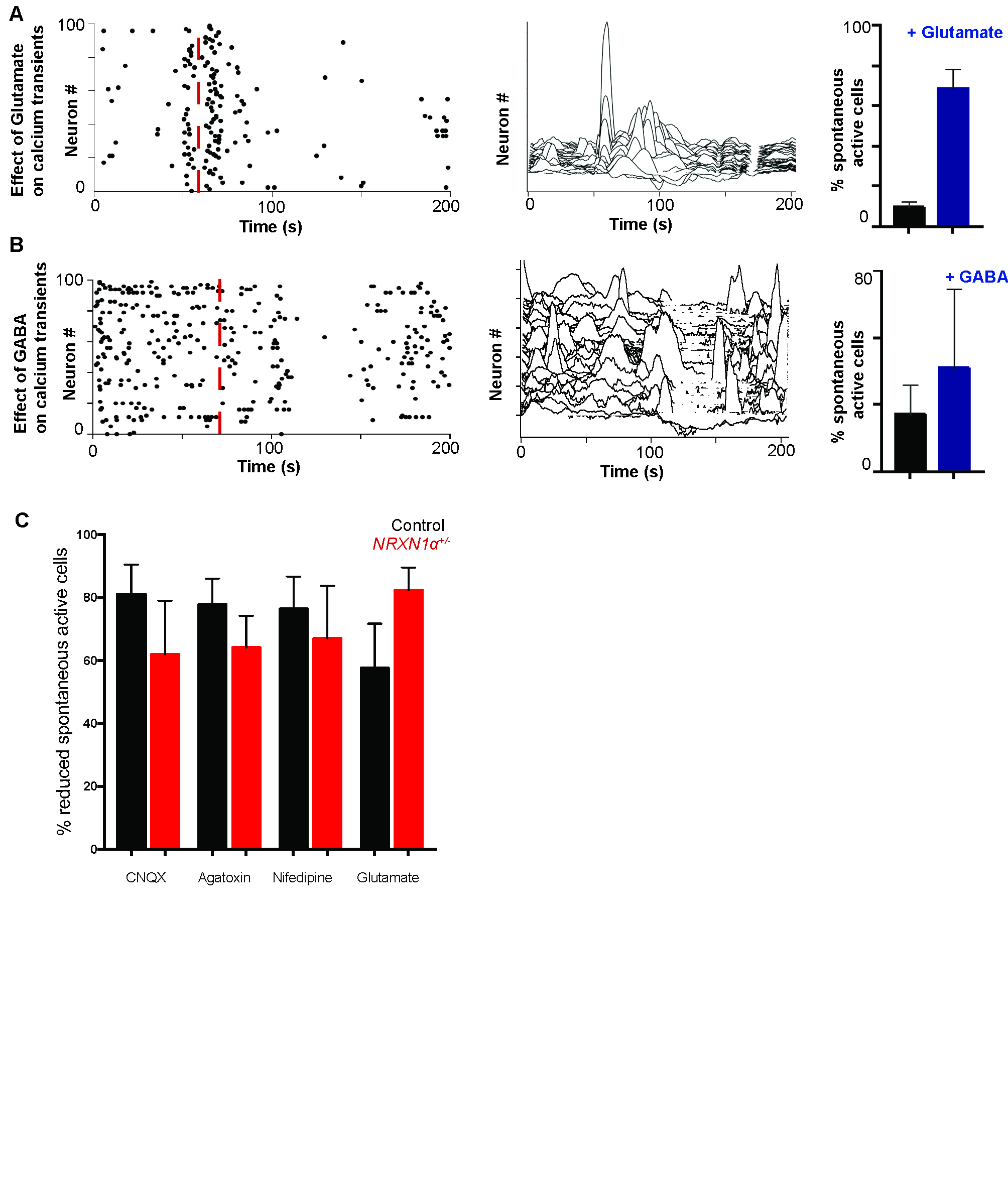


**Figure 6. Spontaneous calcium transients are excitatory dependent.** Representative raster plots (Activity of 100 cells in 200 seconds of recording, each dot represent Ca^2+^ transient activity) waterfall traces (3D representative of calcium transients in 300 seconds over defined threshold level) and their significant change showing the spontaneous Ca^2+^ transient activity of the cells and their response after the application Glutamate (60 uM, A) and GABA (60 uM, B) (n=2-4, 1CC1, 3VCX1, 4CX1,4C3). (C) The percentage of reduced spontaneous calcium transients after the application of CNQX, agatoxin, nifedipine and glutamate on NRXN1α^+/-^ lines (n=2-3, ND1 C1, ND2 C11, ND4-1 C1) in comparison with control lines.


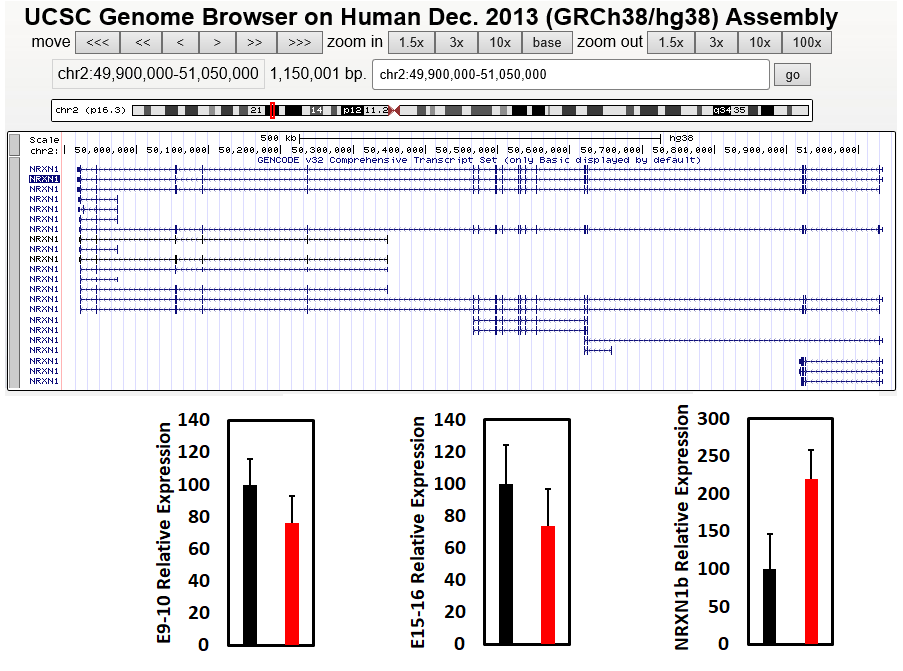

**Figure 7. NRXN1 splicing variants on UCSC database, and** NRXN1α and NRXN1β expression in 100-day iPSC derived neurons. (Top panel) In addition to transcripts transcribed from the NRXN1α (right) and NRXN1β (left) promoters, transcripts also exist to start from the middle of the NRXN1 gene in UCSC database. (Bottom panels) NRXN1α expression was measured by qRT-PCR using primers from exons 9-10 and exons 15-16, respectively, and 24% or 26% reduction was found in NRXN1α^+/-^iPSC derived neurons. The NRXN1β expression on the other hand were increased by 262% in NRXN1^+/-^iPSC derived neurons using NRXN1β-specific exon 18 primers. Data were Mean ± SEM, from 2 independent cortical neuronal differentiation.

**Figure 8. Calcium transients’ properties**. The frequency, duration and amplitude of all “n” number (number of recording) are broken down in all controls and NRXN1^+/-^ lines.

**Figure 9. Comparison of spontaneous calcium transients between ASD (ND1) and a sibling control (1CC1).** The frequency (A, *p*=0.01) and duration (B, *p*=0.02) of calcium transients were significantly increased in ND1 neurons compared to that derived from the sibling control 1CC1.

**Figure 10. Comparison of spontaneous calcium transients of each individual probands to controls showed significant and consistent changes in frequency.** The Frequency, duration and amplitude of each probands were compared to all controls. ND1 and ND4-1 showed significant elevated frequency and amplitude, where ND2 showed significantly increased frequency and duration. Meanwhile, the frequency of calcium transients showed significant difference in all probands (ND1 *p*=0.003, ND2 *p*=0.04, ND4 *p*<0.0001).

**Figure 11. Comparison of controls and ASD with or without seizure.** (A-C) Comparison of controls and ASD with seizure (ND1 and ND4-1) revealed significant changes in the frequency and amplitude of calcium transients. (D-F) ASD without seizure (ND2) showed significant changes in the frequency and duration of calcium transients from the controls. The increase of the frequency was more prominent in two ASD probands with seizure (A) than the ASD without seizure (D). (G-I) However, the ASD with (ND1 and ND4-1) or without seizure (ND2) presented no significant difference on either the frequency, or duration or amplitude of calcium transients. Note the “n” number of the patients were small, with 4 lines from two ASD probands with seizure and 2 lines from one ASD without seizure.


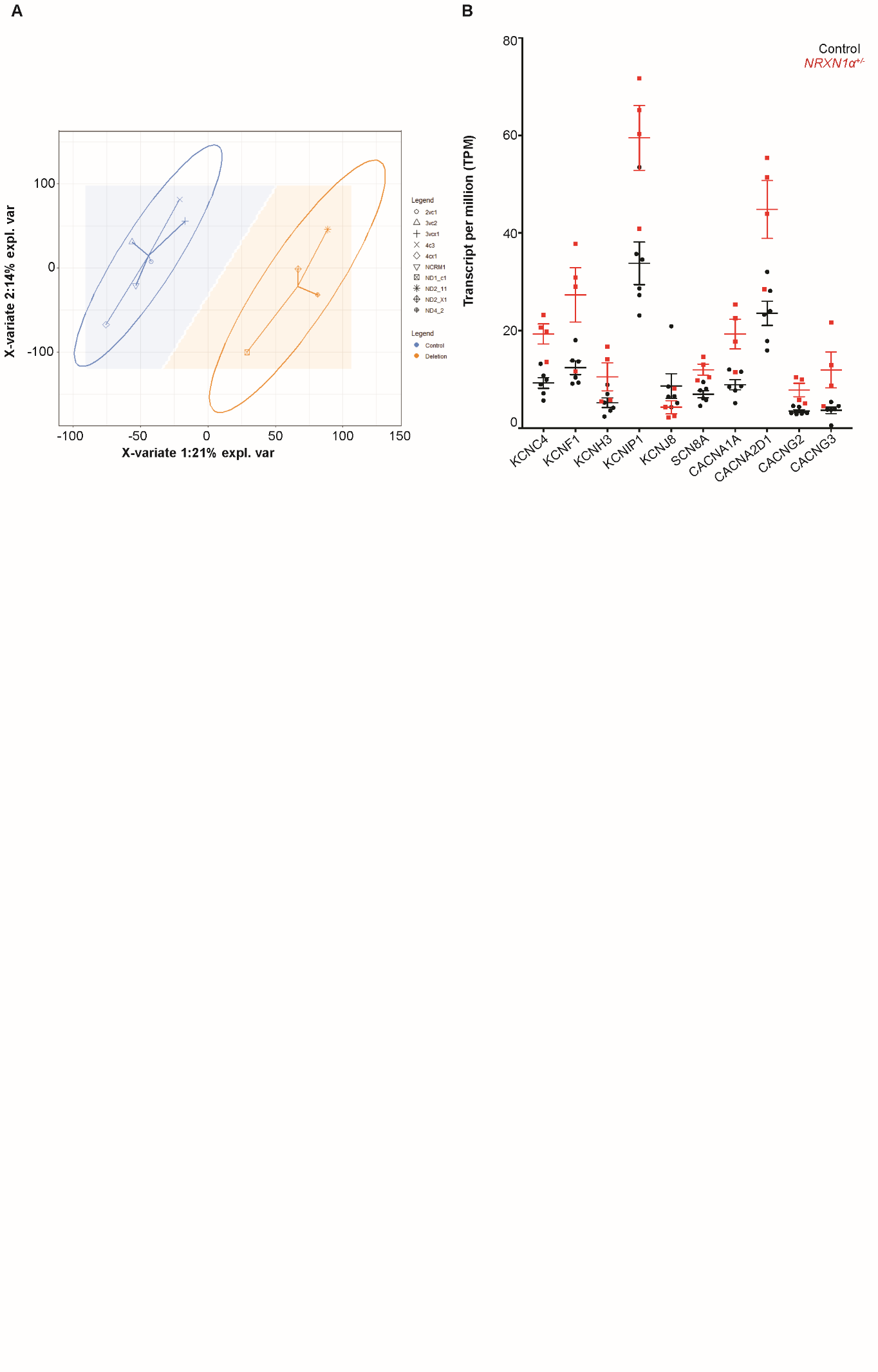


**Figure 12. PLS Discriminant Analysis (PLS-DA) for supervised clustering of RNASeq data and comparison of DEGs encoding ion channels.** (A) The whole genome RNASeq was performed and expressed in TPM (transcript per million). The PLS Discriminant Analysis (PLS-DA) was carried out on the RNASeq data for supervised clustering. Plotting of the 10 RNASeq samples showed consistent clustering into two groups. (B) The comparison of 10 most interactive DEGs (encoding ion channels) with control lines in black and NRXN1α^+/-^ lines in red. All data summary are Mean ± SEM.

**
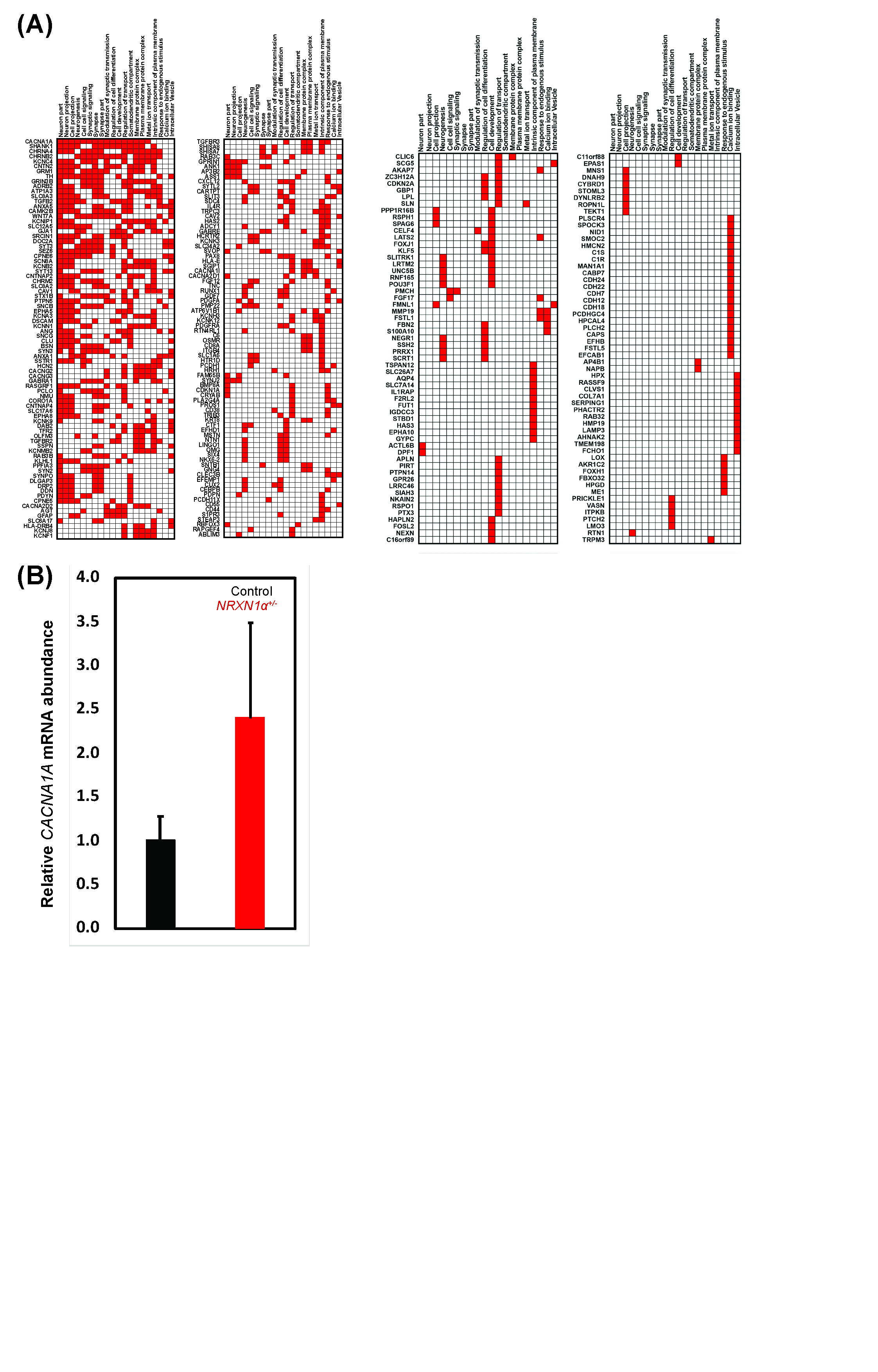
**

**Figure 13. (A) Gene enrichment analysis by GSEA among all biological processes, molecular function and cellular component and validation of CACNA1A expression**. *CACNA1A* was identified as the most overlapped genes among the top 20 pathways with 275 overlapped genes. In the heatmap, the genes appeared in the pathway were filled in red and genes not appeared in the pathways are indicated in white. (B) Relative *CACNA1A* mRNA abundance in control and *NRXN1α+/-* neuronal cultures, 2.4-fold increase in *NRXN1α+/-* neurons detected by qRT-PCR.
